# Identification of Putative Novel Class-I Lanthipeptides in Firmicutes: A Combinatorial *In Silico* Analysis Approach Performed on Genome Sequenced Bacteria and a Close Inspection of Z-Geobacillin Lanthipeptide Biosynthesis Gene Cluster of the Thermophilic *Geobacillus* sp. Strain ZGt-1

**DOI:** 10.3390/ijms19092650

**Published:** 2018-09-06

**Authors:** Rawana N. Alkhalili, Björn Canbäck

**Affiliations:** 1 Biotechnology, Department of Chemistry, Lund University, SE-221 00 Lund, Sweden; 2 Department of Biology, Lund University, SE-221 00 Lund, Sweden; Bjorn.Canback@biol.lu.se

**Keywords:** antimicrobial, antiSMASH, bacteriocins, BAGEL, firmicutes, *Geobacillus*, lanthipeptides, lantibiotics, lantipeptides, Z-geobacillin

## Abstract

Lanthipeptides are ribosomally synthesized and post-translationally modified polycyclic peptides. Lanthipeptides that have antimicrobial activity are known as lantibiotics. Accordingly, the discovery of novel lantibiotics constitutes a possible solution for the problem of antibiotic resistance. We utilized the publicly available genome sequences and the bioinformatic tools tailored for the detection of lanthipeptides. We designed our strategy for screening of 252 firmicute genomes and detecting class-I lanthipeptide-coding gene clusters. The designed strategy resulted in identifying 69 class-I lanthipeptide sequences, of which more than 10% were putative novel. The identified putative novel lanthipeptides have not been annotated on the original or the RefSeq genomes, or have been annotated merely as coding for hypothetical proteins. Additionally, we identified bacterial strains that have not been previously recognized as lanthipeptide-producers. Moreover, we suggest corrections for certain firmicute genome annotations, and recommend lanthipeptide records for enriching the bacteriocin genome mining tool (BAGEL) databases. Furthermore, we propose Z-geobacillin, a putative class-I lanthipeptide coded on the genome of the thermophilic strain *Geobacillus* sp. ZGt-1. We provide lists of putative novel lanthipeptide sequences and of the previously unrecognized lanthipeptide-producing bacterial strains, so they can be prioritized for experimental investigation. Our results are expected to benefit researchers interested in the *in vitro* production of lanthipeptides.

## 1. Introduction

In parallel with the continuously growing problem of bacterial multidrug resistance together with the customer requirements for using natural antimicrobial compounds and food preservatives in food products, there is a growing need for identifying new natural antimicrobial compounds, among which is the family of lanthipeptides.

Lanthipeptides are ribosomally synthesized cyclic peptides, distinguished by the presence of unusual thioether-linked amino acids, lanthionine (Lan) and (2S,3S,6R)-3-methyllanthionine (MeLan) [1]. Lanthipeptides that have antimicrobial activity are known as lantibiotics (for lanthionine-containing antibiotics) [1]. The lanthipeptide is synthesized as a precursor peptide (generally designated as LanA) that undergoes post-translational modifications to produce the bioactive mature lanthipeptide. The modifications involve dehydration of serine (Ser) and threonine (Thr) residues to dehydroalanine (Dha) and dehydrobutyrine (Dhb), respectively, followed by addition of the thiol groups of cysteine (Cys) residues onto Dha and Dhb which results in the formation of thioether cross-links, yielding the Lan and MeLan residues, respectively [1].

Lanthipeptides are classified based on the biosynthetic enzymes involved in the post-translational modifications into four classes [1]. In the current study, we are focusing on class-I lanthipeptides. For a comprehensive overview of the other classes, we recommend that the reader refer to the review by Arnison et al., 2013 [1]. In class-I lanthipeptides, the dehydration is carried out by a dedicated lanthipeptide dehydratase, generally designated as LanB, while the cyclization is carried out by a lanthipeptide cyclase, generally designated as LanC. LanA is composed of a leader peptide and a core peptide [1,2]. The post-translational modifications take place in the core-peptide which gives the bioactive mature peptide [2]. The modified peptide is exported outside the producing cell via a transmembrane ATP-binding cassette (ABC) transporter, generally designated as LanT [2]. The leader peptide is cleaved off either intracellularly or extracellularly by a lanthipeptide-dedicated serine protease (LanP) or by another protease produced by the host cell [2]. The biosynthesis of lanthipeptides is regulated via the two-component response regulatory system; the sensor histidine kinase (LanK) and the cytoplasmic response regulator (LanR). The lanthipeptide-producing cell protects itself against the inhibitory effect of its own lanthipeptide via immunity proteins (LanIEFG) [1] (details are given in Section 2.3.2).

A great deal of attention has recently been directed towards lanthipeptides, thanks to their polycyclic nature which offers them protease-resistance and stability, as well as target-specificity due to their limited conformational freedom, making them superior to many other peptide compounds [3]. Moreover, different research studies have demonstrated evidence of the potential applications of antimicrobially-active lanthipeptides as therapeutic alternatives to traditional antibiotics, as prophylactics in the veterinary applications, as food natural preservatives [4], and also as antimicrobial agents against plant pathogens [5]. The antimicrobial activity of a number of class-I lanthipeptides against different pathogens has been proven. For example; the most well-studied lanthipeptide, nisin, produced by *Lactococcus lactis*, antagonizes the growth of different food-borne pathogens, food-spoiling bacteria [6], and methicillin-resistant *Staphylococcus aureus* (MRSA) strains [7]. Other lanthipeptides have also proved their potent antimicrobial activity. Accordingly, the interest in discovering new lanthipeptides is rising [3], and the increase in the bacterial genomic sequence data deposited in the public databases—through *in silico* analyses and genome-mining strategies—is aiding in finding putative lanthipeptides coded on the genomes of bacterial strains that have not yet been identified as lanthipeptide producers [2].

In addition to the significant potential applications of lanthipeptides, the need for revealing their presence on bacterial genomes also comes from the role the lanthipeptides might be playing in enhancing the pathogenicity of the virulent lanthipeptide-producing bacteria, such as MRSA, as proposed in [8,9]. Production of antimicrobially-active lanthipeptides by pathogens is supposed to have a negative impact on the infected host since lanthipeptide-sensitive microbes maybe out-competed resulting in a growing number of pathogens [8,9]. This indicates that it is critical to achieve further progress in identifying lanthipeptides coded on the bacterial genome, since they could be targeted as a therapeutic intervention in such cases [8,9]. In other words, identification of lanthipeptides coded on the genomes of microorganisms is clinically important, whether for the sake of developing new antibiotics or for the prevention of infection.

Among the significant microorganisms in terms of lanthipeptide production are firmicutes. Members of the phylum Firmicutes are widely distributed in nature. The endospore-forming ones, as bacilli, are able to survive or even thrive in harsh conditions, and have broad metabolic diversity [10]. These features facilitate the establishment of vast industrial and biomedical applications [11]. To date, the majority of the identified antimicrobially-active lanthipeptides are produced by firmicutes [12]. However, we have found that there are still several lanthipeptide-coding genes harbored on the genomes of firmicutes that have been overlooked during the annotation of the original genome data submitted by the research group, inaccurately annotated, annotated as coding for hypothetical proteins, or have been annotated as coding for antibiotics without specifying the type of ‘antibiotic’. We have also observed similar cases with the NCBI Reference Sequence (RefSeq) annotation of certain firmicute genomes. Given the biotechnological potential of firmicutes and our research interest in their antimicrobial potential, especially the thermophilic *Geobacillus*, and due to the fact that firmicutes also include lanthipeptide-producing pathogens, such as staphylococci, we decided to screen the publicly-available genome sequences of firmicutes for lanthipeptide-coding genes.

*In silico* screening for class-II lanthipeptide clusters (lanM genes) was carried out previously and resulted in identification of novel lanthipeptides [13,14]. However, there is only one comprehensive study aimed for the identification of novel class-I lanthipeptides through *in silico* screening of bacterial genome sequences. The identification was carried out by Marsh et al. and the study was published in 2010 [15]. From 2010 until now, the availability of genomic sequence data has dramatically increased, and the genome-mining software tools available for identifying putative lanthipeptide clusters have improved, raising the chance for predicting new putative lanthipeptide-producing bacterial strains. Within this context, antiSMASH (antibiotics and secondary metabolite analysis shell) is a useful software pipeline, which has been accessible for lanthipeptide prediction, among other secondary metabolites—starting from 2011 [16]—and has been greatly improved for lanthipeptide detection and classification starting from 2014 [17]. The antiSMASH software analyzes the genome sequence, detects putative lanthipeptide biosynthetic gene clusters, predicts the post-translational modifications, determines the class of the detected lanthipeptide based on the classification given by Arnison et al., 2013 [1], and predicts the properties of the modified lanthipeptide [18].

At another level, to fulfill the need for discovering novel antimicrobial compounds, the isolation of bacterial strains from extreme habitats constitutes a promising resource. The bacterial diversity in these ecological systems, particularly the untapped ones, is likely to offer a diverse array of novel natural compounds [19].

In the current study, we employed an *in silico*-based approach using different lanthipeptide identification tools to screen firmicute genomes that have the status of ‘complete sequence’ for class-I lanthipeptides. We identified seven putative novel class-I lanthipeptides that have not been recognized as such neither by the research group who submitted the genome, nor by the NCBI prokaryotic genome annotation pipeline (PGAP) used for annotating the RefSeq genome records (Table 1). Moreover, these putative lanthipeptides are not identical to any of the known lanthipeptides, and some of them do not even show any homology to known lanthipeptides. We also identified lanthipeptides coded on genomes of bacterial strains that have not been recognized as lanthipeptide producers. Furthermore, we are paying close attention to the thermophilic *Geobacillus*, which is seen as a genus with a great biotechnological potential [20].

We are proposing the thermophilic bacterium *Geobacillus* sp. strain ZGt-1, which we have isolated earlier from Zara hot spring in Jordan [21,22] as a producer of a putative class-I lanthipeptide, which we term herewith as Z-geobacillin, an analog of geobacillin I.

## 2. Results and Discussion

### 2.1. Lanthipeptide Identification Workflow

**Figure 1:**
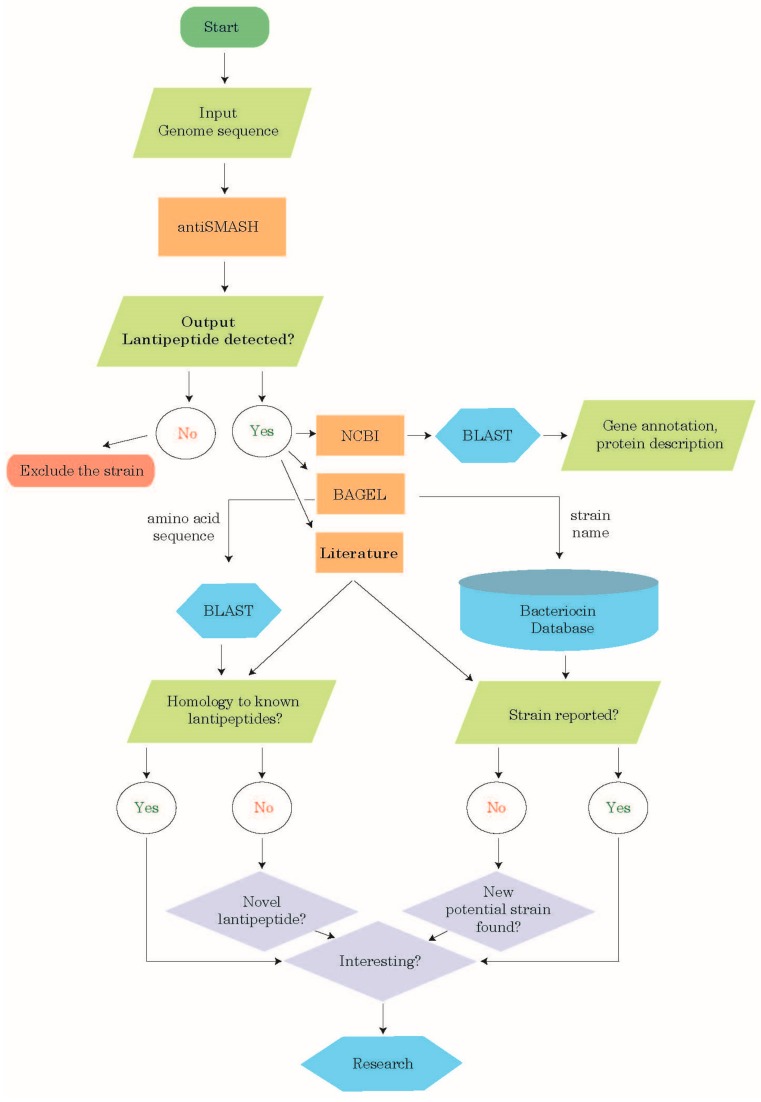
Workflow chart summarizing the analyses steps carried out in this study.

The strategy we plotted for identifying class-I lanthipeptides started with downloading the complete genome sequences of firmicute bacterial strains deposited in the RefSeq genome database (Figure 1). This was followed by screening the genome sequences for class-I lanthipeptide clusters using the latest version of antiSMASH (antiSMASH 4.0.2; hereafter referred to as antiSMASH, except for when a context of comparison between different versions of the software is set, then the version number is stated, as discussed below) [23]. This version of antiSMASH analyzes the genome sequence and identifies the gene coding for the lanthipeptide precursor using an algorithm from RODEO (Rapid ORF Description and Evaluation Online) genome-mining platform. The utilized robust algorithm employs a combination of the machine-learning algorithm and lanthipeptide motif analysis. In certain cases, in addition to applying antiSMASH 4, we used the older version of antiSMASH (antiSMASH 3.0.4; hereafter referred to as antiSMASH 3) [24]. After retrieving the amino acid (aa) sequences of the predicted lanthipeptides, we analyzed them using blastp and tblastn [25].

In addition to the NCBI BLAST analysis, we conducted bacteriocin genome mining tool (BAGEL) BLAST analysis [26] of the antiSMASH-predicted lanthipeptide sequences against BAGEL4 class-I and class-II bacteriocin databases (Figure 1). This step aimed at evaluating whether the predicted lanthipeptide was homologous to previously characterized bacteriocins reported in BAGEL4 databases, or represented a putative novel lanthipeptide, and thus, an attractive target for further research. To identify bacterial strains that have not been previously described as lanthipeptide producers, we referred to BAGEL4 databases [26], where we checked whether the strain, whose genome sequence was predicted by antiSMASH to harbor a class-I lanthipeptide cluster(s), had been previously reported as a producer of bacteriocins, or not yet. We have also referred to the literature to complement the information in BAGEL databases. In some cases, in addition to using antiSMASH, we applied BAGEL4 genome mining tool [26].

For graphical antiSMASH output of the lanthipeptide biosynthesis gene clusters of each of the genomes, please see (http://130.235.46.10/Lanthipeptides/) (last checked on the 5 September 2018).

### 2.2. Identification of Putative Novel Lanthipeptides

Bacteriocin-coding-genes can be overlooked by the automated genome annotation tools due to their short open reading frames (ORFs) [27], making it difficult to have a complete bacteriocin database [28]. Therefore, even in case of accurate detection of the short ORF, it is not necessarily possible to identify the gene product as a bacteriocin [29]. The gene product could instead be annotated as a protein of unknown function, or as a hypothetical/uncharacterized protein [29]. These proteins could represent putative novel bacteriocins yet to be identified. The difficulties in the detection of bacteriocins call for carrying out a continuous and thorough analysis of different microbial genome sequences.

In the current study, the resulting antiSMASH-predicted lanthipeptide data set was composed of 69 aa sequences (Table S1). To identify the putative novel lanthipeptides among them, we adopted the following criterion; a given antiSMASH-predicted lanthipeptide is identified in the present study as putative novel when it has not been previously reported or identified as a lanthipeptide. Accordingly, a lanthipeptide predicted in the current study is described as putative novel when it fulfills all of the following conditions: (1) its coding gene was not annotated or annotated in the original genome record, which was submitted by the research group who sequenced the genome and deposited it in NCBI GenBank, as coding for a hypothetical/uncharacterized protein; (2) its coding gene has not been annotated or annotated in the RefSeq genome record, which was annotated by the NCBI PGAP, as coding for a hypothetical/uncharacterized protein; (3) BAGEL BLAST of the aa sequence of the predicted lanthipeptide returned protein hit(s) with less than 100% identity to the characterized small bacteriocins deposited in BAGEL databases; and (4) the predicted lanthipeptide has not previously been highlighted in literature studies, whether that study was based on an *in silico* analysis of the genome analyzed in our study, or based on an experimental investigation of the predicted lanthipeptide. As a result, we identified seven putative novel lanthipeptides.

In addition to the 69 identified lanthipeptides, we propose the lanthipeptide Z-geobacillin predicted to be produced by the novel *Geobacillus* sp. strain ZGt-1 which we isolated from Zara hot spring in Jordan [21,22] as a putative novel lanthipeptide (Table 1; Table S1), and hereby we report it and describe its biosynthesis gene cluster.

Accordingly, out of the resulting set of antiSMASH-predicted lanthipeptides, we identified eight putative novel lanthipeptides in total (Table 1). Among the producers of these putative novel lanthipeptides, there was one species that has not been reported in the literature as a lanthipeptide producer. We also identified 40 bacterial strains that have not been experimentally investigated as potential lanthipeptide producers (Table 2). 

### 2.3. Class-I Lanthipeptide Biosynthetic Gene Clusters in Firmicutes

**Figure 2:**
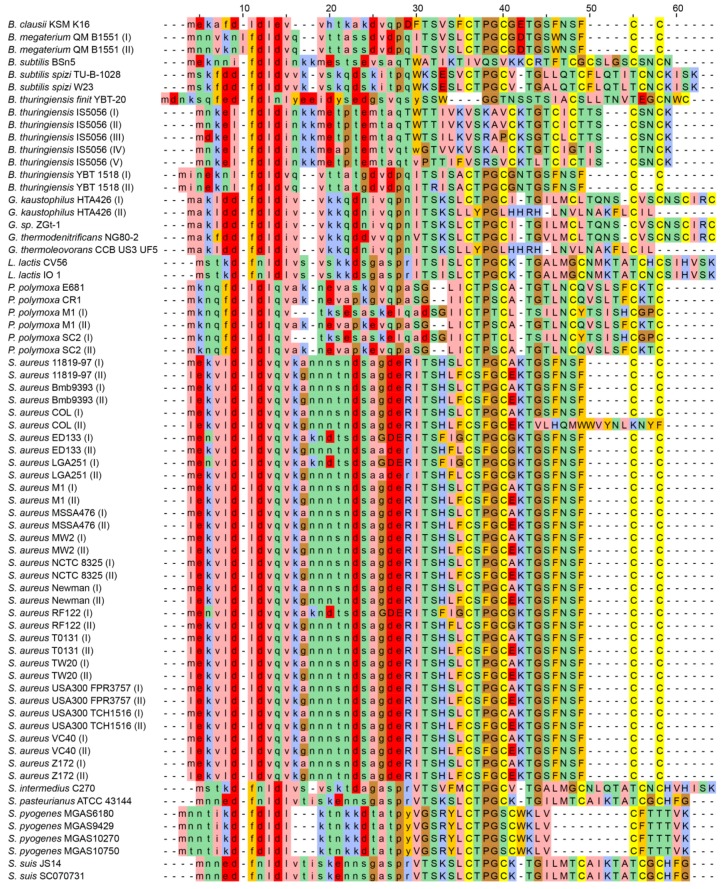
Alignment of all identified class-I LanA precursors with the exception of the lanthipeptide of *Streptococcus intermedius* strain B196 which aligned poorly. Leader peptides are in lower case and are reported as shown in the results of antiSMASH. No manual editing of the alignment was performed. For the full name of bacterial species, see Table S1.

The identified lanthipeptide producers among firmicutes are discussed below in alphabetical order, and the alignment of these lanthipeptides is illustrated in Figure 2. In the figure, aa abbreviations in the leader peptides are in lowercase while the core peptides are in uppercase. As seen in the alignment, the boundary between leader and core peptides is probably not always correctly predicted by antiSMASH; for example, the arginine (R) in position 29. The core peptides are rich in cysteine residues, but also—to a lesser extent—in serine and threonine residues, which constitute a prerequisite for the thioether cross-links to form. Not immediately recognized, since about half of the sequences derive from *Staphylococcus aureus* strains, is that the homology between sequences from different species is quite low. Therefore, identification of lanthipeptides cannot solely rely on sequence homology.

#### 2.3.1. Identification of *Bacillus*-associated Lanthipeptide Gene Clusters

Bacterial members of the genus *Bacillus* belong to the class ‘Bacilli’, order Bacillales, family Bacillaceae [40]. They are Gram-positive, motile or non-motile, aerobic or facultative anaerobic, and are spore-formers [40]. They can be isolated from different ecological niches, such as soil, water, food, and clinical samples [40]. The *Bacillus* genus represents a rich source of antimicrobial compounds, including lanthipeptides with a broad spectrum of inhibitory activity [32,41]. Below, we are presenting strains of different *Bacillus* species coding for class-I lanthipeptides.

• *Bacillus clausii* KSM-K16

Different strains of *B. clausii* have been described as probiotics, as they demonstrate antimicrobial and immunomodulatory activities [42]. A class-I lanthipeptide with antimicrobial activity; clausin, has been isolated from a strain of *B. clausii* [43]. Our analysis indicated that this strain produces a class-I lanthipeptide other than clausin, and this is in agreement with the RefSeq annotation. Please refer to S.2.3.1.1 in the Supplementary Material file for a full description of the analysis. To our knowledge, the potential of strain KSM-K16 as a lanthipeptide producer has not been experimentally investigated so far (Table 2).

• *Bacillus megaterium* QM B1551

*B. megaterium* is the largest species among all bacilli in terms of cell size [44]. The antiSMASH analysis indicated that the chromosomal DNA of strain QM B1551 does not code for lanthipeptides. On the other hand, the strain has seven plasmids, and our analysis indicated that plasmid pBM700 harbors a class-I lanthipeptide cluster which has two putative genes coding for two identical class-I lanthipeptides. The antiSMASH results are in agreement with the RefSeq annotation and with Xin et al., 2015 [31] (details are given in S.2.3.1.2). To our knowledge, the potential of this strain as a lanthipeptide-producer has not been experimentally characterized (Table 2).

• *Bacillus subtilis*

*B. subtilis* strains produce a variety of antimicrobial compounds, including different kinds of lanthipeptides as reviewed in [45]. Below, we are presenting three strains as (potential) producers of class-I lanthipeptides.

*B. subtilis* BSn5

Strain BSn5 is an endophytic bacterium of the plant *Amorphophallus konjac* [33]. The strain harbors a class-I lanthipeptide biosynthetic gene cluster coded on the chromosomal DNA, and has not been experimentally investigated for lanthipeptide production (Table 2).

The nucleotide (nt) sequence of the gene coding for the antiSMASH-predicted lanthipeptide (Table S1) is 100% identical to ‘BSN5_RS12800’ which codes for a hypothetical protein in the RefSeq record (Table S2). Deng et al., 2011 who sequenced the genome of strain BSn5 noted the presence of the gene and described it as paenibacillin-like lanthipeptide [33]. Phelan et al., 2013 briefly mentioned that strain BSn5 has a putative lanthipeptide-coding gene and identified the lanthipeptide as subtilomycin [32]. Thus, these results support our analysis (Table S6). BAGEL BLAST indicated that the predicted lanthipeptide is 100% identical to subtilomycin (Table 2; Table S2).

*B. subtilis spizizenii* DSM 15029T (TU-B-10)

Strain DSM 15029 (also known as TU-B-10) was isolated from soil in Tunisia [46], and is the type strain of *B. subtilis* subsp. *spizizenii*, as indicated in the German Collection of Microorganisms and Cell Cultures (DSMZ). The strain is known for producing entianin, a class-I lanthipeptide [47]. The antiSMASH results are in agreement with this fact and with the RefSeq annotation (details are given in S.2.3.1.3).

*B. subtilis spizizenii* W23

Strain W23 is the best characterized strain of *B. subtilis spizizenii* [48]. However, to our knowledge, it has not been investigated for the production of bacteriocins (Table 2). Our analysis indicated that strain W23 has the potential to produce a class-I lanthipeptide, and this is in agreement with the RefSeq annotation (details are given in S.2.3.1.4).

• *Bacillus thuringiensis*

*B. thuringiensis* strains are widely spread in nature; they have been isolated from soil, insects, and plant leaves [49]. They are used as natural biopesticides due to the production of toxic proteinaceous crystals [50,51]. Some strains of *B. thuringiensis* are also known for the production of peptides with antibacterial, antifungal, and anticancer activities, as reviewed in [52].

*B. thuringiensis* serovar finitimus YBT-020

This strain represents one of only a few examples of *B. thuringiensis* strains with a special phenotype of the toxic crystals [53] (for details, the reader is recommended to refer to [51,53]). The lanthipeptide production potential of strain YBT-020 has not been investigated (Table 2).

Our analysis indicated that the strain has a putative class-I lanthipeptide cluster, and it also showed that the gene coding for the antiSMASH-predicted lanthipeptide has not been annotated in the RefSeq record (Table S2). On the other hand, the gene has been annotated on the original genome. While the nt sequence of the gene coding for the predicted lanthipeptide (Table S1) is 100% identical to ‘YBT020_05970’ annotated on the original genome as coding for a hypothetical protein (Table S2), the last nt in the stop codon of ‘YBT020_05970’ (i.e., A in TAA) has not been reported by antiSMASH 4. Accordingly, the reported positions of the coding gene do not match those of ‘YBT020_05970’. In order to resolve the correct position of the antiSMASH-predicted gene, and thus, its nt sequence, we analyzed the genome of the strain using antiSMASH 3. The position of the gene was identical to that of ‘YBT020_05970’ where no nt was missing from the antiSMASH 3–predicted gene. Therefore, we corrected the gene position and presented it in Table S1.

InterPro analysis did not identify a lanthipeptide-related domain in the protein. In order to inspect the antiSMASH prediction, we checked the nearby genes in the genome records for the presence of the genes coding for lanthipeptide modifying enzymes. In the original genome, there is a LanB-coding gene annotated as ‘YBT020_05955’ which is upstream of the putative lanthipeptide coding gene ‘YBT020_05970’. However, this protein has been annotated in the RefSeq genome record as a hypothetical protein. Analyzing the aa sequence of this protein using InterPro confirmed its identity as a LanB. A LanC protein coding-gene is also annotated in the original genome record as ‘YBT020_05980’ downstream of ‘YBT020_05970’, but it has been annotated as a hypothetical protein in the RefSeq genome record. InterPro analysis confirmed its identity as a LanC. The presence of these two lanthipeptide modifying enzymes makes it likely that the short ‘hypothetical protein’, which is coded by ‘YBT020_05970’ and predicted as a lanthipeptide by antiSMASH (Table S1), is a lanthipeptide. Furthermore, BAGEL4 genome mining tool as well identified the existence of a class-I lanthipeptide cluster and confirmed the presence of LanB and LanC. BAGEL BLAST indicated that the predicted lanthipeptide did not have any hits to any known small bacteriocins (Table 2). These results indicate that the predicted lanthipeptide could be novel (Table 1) and of interest to be explored. We recommend considering the RefSeq genome record of this strain for re-annotation, and we suggest annotating ‘YBT020_05970’ at the position presented in the original genome as well as by antiSMASH 3 (Table S1).

*B. thuringiensis* serovar IS5056

*B. thuringiensis* serovar IS5056 was isolated from soil in Poland, and is an effective biopesticide [50]. In addition, strain IS5056 has the potential to produce class-I lanthipeptides, as discussed below.

The analysis using antiSMASH indicated that the chromosome of the strain does not possess genes coding for lanthipeptides. On the other hand, the plasmid (pIS56-233) of the strain harbors five putative class-I lanthipeptide-coding genes clustered together. This is a rare case for a lanthipeptide gene cluster, since clustered lanthipeptide-coding genes rarely exceed two [31]. The nt sequences of the first two putative coding genes (H175_233p095; H175_233p096) are identical (Table S1), indicating a gene duplication. These two genes have been annotated as coding for hypothetical proteins on the RefSeq genome (Table S2). However, in addition to our analysis, literature studies suggest these proteins to be lanthipeptides, as discussed below. The other three putative coding genes—H175_233p097 coding for lanthipeptide III, H175_233p098 coding for lanthipeptide IV, and H175_233p099 coding for lanthipeptide V—do not share identical nt sequences (Table S1) and are also predicted to code for hypothetical proteins (Table S2). Strain IS5056 has also been *in silico*-predicted by Xin et al., 2015 as a lanthipeptide-producer using BAGEL3 genome mining tool [31], which supports our results (Table S6).

We noticed that the aa sequences of the predicted five lanthipeptides of strain IS5056 are identical to those of the thuricin 4A biosynthetic gene cluster, coded on the chromosome of *B. thuringiensis* serovar *thuringiensis* strain T01001 [31]. The antibacterial activity of the thuricin 4A cluster was verified by Xin et al., 2015, and ascribed to only one of its four coded lanthipeptides, named as thuricin 4A-4 [31]. The aa sequence of thuricin 4A-4 is identical to the duplicated lanthipeptides coded on the genome of strain IS5056 (Table 2; Table S2). Therefore, an investigation of the antibacterial activity of the five-lanthipeptide coding gene cluster in strain IS5056 should be of interest, especially when *B. thuringiensis* serovar IS5056 has not been experimentally investigated as a producer of class-I lanthipeptides (Table 2). We noticed that thuricin 4A-4 has not been reported in BAGEL databases.

*B. thuringiensis* YBT-1518

Although *B. thuringiensis* is a known producer of antibacterial peptides, to our knowledge, the potential of strain YBT-1518 as a lanthipeptide producer has not been experimentally characterized (Table 2). The antiSMASH analysis indicated that strain YBT-1518 harbors a class-I lanthipeptide biosynthetic gene cluster. This is in agreement with the RefSeq annotation (details are given in S.2.3.1.5).

#### 2.3.2. Identification of *Geobacillus*-associated Lanthipeptide Gene Clusters

Bacterial members of the genus *Geobacillus* belong to the class Bacilli, order Bacillales, and family Bacillaceae. Little is known about lanthipeptides from thermophilic bacteria in general, and the genus *Geobacillus* in particular, with only two lanthipeptides—geobacillin I and geobacillin II, produced by *Geobacillus thermodenitrificans* NG80-2—have been characterized [34]. Investigating the lanthipeptide potential of thermophiles is of significant interest. Although nisin, produced by mesophilic lactic acid bacteria, retained its antibacterial activity at pH 2 when autoclaved at 121 °C, it lost its activity when it was at pH 11 and heated at 63 °C for 30 min (reviewed in [6]). Lanthipeptides from thermophiles are expected to be more stable, as demonstrated by Garg et al., 2012 [34]. In the current study, we are presenting three potential class-I lanthipeptide-producing geobacilli. The graphical output of the lanthipeptide biosynthesis gene clusters of the presented geobacilli genomes, and all other genomes as well, is available online (http://130.235.46.10/Lanthipeptides/) (last checked on the 5 September 2018).

• *Geobacillus kaustophilus* HTA426

*G. kaustophilus* HTA426 is a thermophilic bacterium that was isolated from the deep-sea sediment of the Mariana Trench [54]. The strain harbors a class-I lanthipeptide cluster coding for two different lanthipeptides. The antiSMASH results of lanthipeptide (I) are in agreement with the RefSeq annotation and with Garg et al., 2012 [34] (details are given in S.2.3.2.1). To our knowledge, the lanthipeptides of *G. kaustophilus* have not been produced *in vitro* (Table 2).

The nt sequence of the gene coding for the predicted lanthipeptide (II) (Table S1) is 100% identical to that annotated on the original genome as GK0924, coding for a lanthipeptide precursor. This in turn supports our results (Table S6). On the other hand, the gene was inaccurately annotated on the RefSeq genome. The positions of the coding gene indicated by tblastn analysis did not match the positions reported by antiSMASH 4 and the RefSeq. The reported positions were identical to the positions reported for ‘GK_RS01950’ that is longer than the one that should code for the antiSMASH-predicted lanthipeptide which is 50 aa in length. To resolve this issue, we analyzed the genome of the strain using antiSMASH 3 as it annotates the genome independently of the RefSeq annotation, using Prodigal. antiSMASH 3 retrieved a lanthipeptide that is identical to the one retrieved by antiSMASH 4 (50 aa), but with different gene positions (i.e., gene length) that correctly corresponded to a 50-aa lanthipeptide and agreed with the positions presented by tblastn. BAGEL4 genome mining tool detected the presence of a class-I lanthipeptide cluster in the strain but did not identify *lanA*. To confirm that the prediction of the lanthipeptide by antiSMASH 3 and antiSMASH 4 was correct, we analyzed the aa sequence for its protein family. InterPro confirmed that the predicted peptide is a lanthipeptide. Therefore, we suggest annotating the lanthipeptide-coding gene on the RefSeq genome at the detected position presented in (Table S1). As inferred from BAGEL BLAST, the predicted lanthipeptide (II) has 79% identity to geobacillin I (Table 2). Our results agree with the findings of Marsh et al., 2010, who reported the potential of strain HTA426 to produce lanthipeptides I and II [15] (Table S6).

• *Geobacillus* sp. ZGt-1

*Geobacillus* sp. strain ZGt-1 is a thermophilic bacterial strain which we earlier have isolated from Zara hot spring in Jordan [21,22]. Since exploring novel repositories of microorganisms is recommended as it could lead to the identification of novel metabolites [55], we investigated the antimicrobial potential of strain ZGt-1. The strain demonstrated antibacterial activity against a strain of the food spoiling bacterium *G. stearothermophilus*, and against the mesophilic bacterium *B. subtilis* and the pathogenic Gram-negative bacterium *Salmonella typhimurium* [22]. We have confirmed the ability of strain ZGt-1 to produce antimicrobial proteins using culture-based methods and tandem mass spectrometric analysis [22].

Moreover, we analyzed the genome sequence of strain ZGt-1 in order to identify antimicrobial peptide-coding genes. The analysis indicated the potential of ZGt-1 to produce bacteriocins, including a lanthipeptide [21]. Since lanthipeptides produced by thermophilic bacteria are of interest due to the reasons mentioned above together with the fact that microbiota of hot springs have not been investigated for their potential as lanthipeptide producers, we decided in the current study to take a closer look at the lanthipeptide coded on the chromosome of strain ZGt-1, as a first step towards characterizing it.

• Architecture of the Z-geobacillin lanthipeptide gene cluster in *Geobacillus* sp. strain ZGt-1

**Figure 3:**
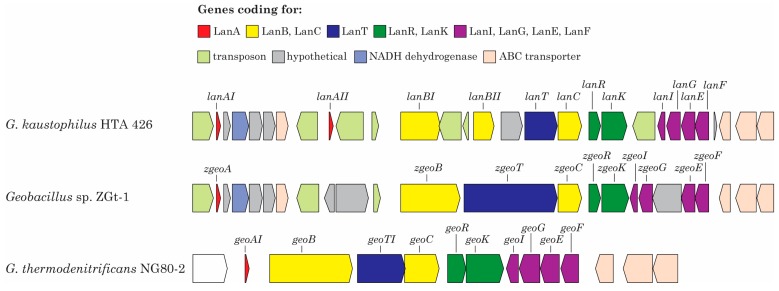
Class-I lanthipeptide clusters of three *Geobacillus* strains, *G. kaustophilus* HTA426, *Geobacillus* sp. ZGt-1, and *G. thermodenitrificans* NG80-2. The illustrated clusters were drawn after the lanthipeptide biosynthesis gene clusters generated by antiSMASH 3, which was run by analyzing the genome FASTA files of the strains [24]. The locus-tags of the genes and accession numbers of the coded proteins are presented in Tables S3–S5 for strains *Geobacillus* sp. ZGt-1, *G. kaustophilus* HTA426, and *G. thermodenitrificans* NG80-2, respectively.

Analysis of the chromosome genome sequence (LDPD01000000) of *Geobacillus* sp. strain ZGt-1 using antiSMASH indicated that the strain harbors a class-I lanthipeptide biosynthesis gene cluster. The cluster contains all the machinery genes commonly found in clusters of this class of lanthipeptides, as reviewed in [2,56]. The cluster contains a short ORF (171 base pairs), designated here as *zgeoA*, coding for the precursor peptide on the forward DNA strand (Table S1), on contig 6_34 (Table S3). The genes required for the biosynthesis, modification, and transport of Z-geobacillin, as well as the self-immunity protein-coding genes are located downstream of *zgeoA* (Figure 3)*.* These genes are a lanthipeptide dehydratase-coding gene (*zgeoB*), a lanthipeptide cyclase-coding gene (*zgeoC*), ABC transporter-coding gene (*zgeoT*), a two-component transcriptional regulator; sensor histidine kinase-coding gene (*zgeoK*) and response regulator-coding gene (*zgeoR*), and self-immunity protein-coding genes *zgeoI*, *zgeoG*, *zgeoE*, and *zgeoF* (Figure 3)*.* However, the cluster does not contain a gene coding for a lanthipeptide protease (LanP) to remove the leader peptide. Similar cases were reported by Garg et al., 2012 for different strains of *Geobacillus* [34]. Proteolytic removal of the leader peptide may be carried out by another protease coded anywhere on the genome of the producing bacterium, as previously reported for subtilin produced by *B. subtilis* [57].

**Figure 4:**
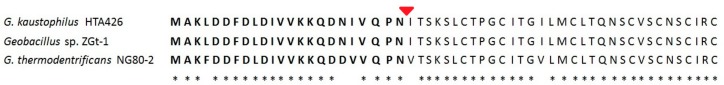
Amino acid sequence alignment of the class-I lanthipeptides of *G. kaustophilus* HTA426 (lanthipeptide I), *Geobacillus* sp. ZGt-1 (Z-geobacillin), and *G. thermodenitrificans* NG80-2 (geobacillin I). The leader peptides are typed in bold as opposed to the core peptides. (*) denotes a conserved aa in the three strains. The red arrow indicates the (antiSMASH-predicted) proteolytic cleavage site for the removal of the leader peptide. The alignment was performed using BioEdit [58].

The putative lanthipeptide precursor—ZgeoA—is composed of 56 aa residues (Figure 4; Table S1). The leader peptide (23 aa) is positioned at the N-terminal region of ZgeoA (Figure 4; Table S1). Usually, the leader peptide is cleaved off in the final step of biosynthesis by a protease [34]. Based on the antiSMASH prediction, the site of the proteolytic cleavage is ProAsn↓Ile (PN↓I) (Figure 4). The core peptide at the C-terminus of ZgeoA has 33 aa residues (Table S1), and it is this part that undergoes post-translational modifications which, with the leader peptide removal, should give the final bioactive Z-geobacillin. Since not all Ser/Thr residues necessarily undergo dehydration, antiSMASH predicted alternative molecular weights of Z-geobacillin based on different probabilities of the number of Ser/Thr residues expected to be post-translationally modified to form Dha/Dhb, respectively [18]. The predictions gave 3292.0 Da (assuming that all the nine Ser and Thr residues in the core peptide are dehydrated), 3310.0 Da (assuming that one of these residues is not dehydrated), or 3328.0 Da (assuming that two of the residues are not dehydrated). There are seven Cys residues in the core peptide of ZgeoA expected to be involved in Lan or MeLan bridges with Dha or Dhb residues; therefore, the thioether (Me)Lan bridges in the core peptide are predicted to be seven. The bridges confer conformational rigidity and stability to the lanthipeptide [34].

As indicated by BLAST analysis, Z-geobacillin shows 100% identity over its entire length to lanthipeptide (I) of *G. kaustophilus* HTA426 (Figure 4), but it is 91% identical to geobacillin I (Table 2). As shown in Figure 4, the different residues between the sequences of geobacillin I and Z-geobacillin, are synonymous as they share similar chemical properties, with the differences being more frequent in the leader peptide. These differences come in the form of having Leu (L) at position 4 in Z-geobacillin versus Phe (F) in geobacillin I, Asn (N) at position 18 versus Asp (D), and Ile (I) at position 19 versus Val (V) (Figure 4). On the other hand, the core peptide has differences at positions 1 and 15 where there are Ile (I) residues in the core of Z-geobacillin versus Val (V) in geobacillin I (Figure 4). As in the case of Z-geobacillin, thioether bridges in geobacillin I are also seven, as predicted by antiSMASH and as reported by Garg et al., 2012 [34]. The same number of bridges was predicted by antiSMASH to form in the putative core peptide of lanthipeptide (I) of *G. kaustophilus* HTA426 as well. Interestingly, Garg et al., 2012 proved that geobacillin I was more stable than nisin at pH 7 and 8, at temperatures 37 and 60 °C [34]. In addition to the thermophilic nature of *Geobacillus*, the stability of geobacillin I can be ascribed to the larger number of thioether bridges (seven) compared to those in nisin (five) [34]. Since Z-geobacillin is also predicted to form seven thioether bridges, it is expected to have a similar stability as geobacillin I.

The putative proteins coded by the Z-geobacillin gene cluster and predicted as ZgeoB, ZgeoC, and ZgeoT are 99% identical to those associated with the lanthipeptides of *G. kaustophilus* HTA426 and *G. thermodenitrificans* NG80-2. LanR is 100% identical to that coded by *G. kaustophilus* but only over part of its length. As inferred by antiSMASH analysis and also indicated in [15], *G. kaustophilus* strain has two putative lanthipeptide precursors and two putative LanB coded by its lanthipeptide gene cluster (Figure 3). On the other hand, one of each of these protein types is coded by the gene cluster of Z-geobacillin and that of geobacillin I. Moreover, the putative cluster of Z-geobacillin has genes predicted to code for transposases and hypothetical proteins, and these genes are inserted between the predicted lanthipeptide-associated genes (Figure 3). However, the insertions in the putative Z-geobacillin gene cluster are less frequent than those found in the cluster of *G. kaustophilus* HTA426 (Figure 3). Consequently, the positions of the predicted genes in the putative clusters of strain ZGt-1 and HTA426 are not the same (Figure 3). On the contrary, the geobacillin I gene cluster is condensed and has no inserted genes (Figure 3). Thus, even with the (almost) identical precursor peptide in the three strains, the biosynthetic gene clusters are not identical. It is worth mentioning that strain ZGt-1 and strain HTA426 were isolated from aquatic environments; from Zara hot spring and from the deep-sea sediment of the Mariana Trench [54], as discussed above, respectively, unlike *G. thermodenitrificans* NG80-2 which was isolated from Dagang oil fields in China [59].

The antibacterial activity of geobacillin I has been experimentally verified [34]. It has been proven active to different extents against different Gram-positive bacteria [34]. Geobacillin I has been more active than nisin against *Streptococcus dysgalactiae* ATCC 27957 which causes bovine mastitis [34]. These features of geobacillin I support the possibility that Z-geobacillin is a putative antibacterially-active lanthipeptide.

• Putative Biosynthesis Pathway of Z-Geobacillin Lanthipeptide in Strain ZGt-1

**Figure 5:**
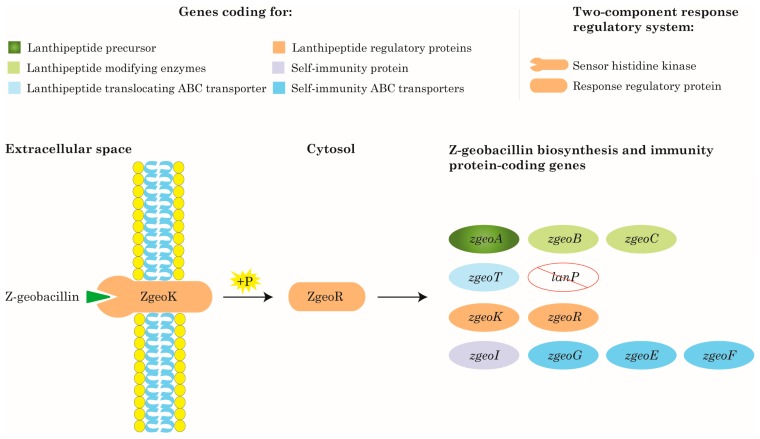
Schematic illustration of the putative biosynthesis pathway of Z-geobacillin. Amino acid sequences of the *in silico*-predicted proteins of strain ZGt-1 were annotated using the pathway mapping tool available from KEGG Automatic Annotation Server (KAAS) [61]. Predicted pathway indicated that the strain has all the genes required for the lanthipeptide biosynthesis, regulation and self-immunity, except for a dedicated protease-coding gene (represented as a white oval shape with a crossing red line). Details of the biosynthesis pathway are given in the text. The scheme was re-drawn after the KAAS-generated lanthipeptide biosynthesis pathway map of strain ZGt-1. Identical colors reflect correlated biological functions.

The organization of the lanthipeptide genes in clusters makes it easy to deduce the biosynthetic pathway [1]. The lanthipeptide biosynthesis pathway, or post-ribosomal peptide synthesis as referred to by Arnison et al., 2013 [1], follows a general model [60]. Due to the *in silico* detection of all the crucial class-I lanthipeptide biosynthetic genes in the genome of ZGt-1, we expect the strain to have a complete biosynthetic pathway. This was confirmed when the lanthipeptide biosynthetic pathway of strain ZGt-1 was evaluated using the Kyoto Encyclopedia of Genes and Genomes (KEGG) Automatic Annotation Server (KAAS) [61] (Figure 5).

We suggest a model of the biosynthesis pathway of Z-geobacillin and the regulation of its production. Previous research suggests that the model will behave as described below.

The biosynthesis pathway of Z-geobacillin is expected to start with the translation of the putative gene; *zgeoA* coding for precursor peptide ZgeoA which consists of leader and core peptides. Dehydration of Ser and Thr residues in the core peptide by the putative lanthipeptide dehydratase ZgeoB will result in the formation of Dha and Dhb residues, respectively. This will be followed by the cyclization reaction, catalyzed by the putative lanthipeptide cyclase ZgeoC, where the thioether cross-links will be formed. The modified peptide is expected to be exported outside the producing cell via the putative transmembrane ABC transporter, ZgeoT. The lanthipeptide remains inactive until the cleavage of the leader peptide, which usually takes place either extracellularly after exporting the modified peptide or intracellularly before translocation by the ABC transporter [2,62]. As mentioned above, in case of Z-geobacillin, this proteolytic cleavage is expected to be carried out by a protease coded elsewhere on the genome of strain ZGt-1, as inferred from [34,57].

In principle, the regulation of lanthipeptide biosynthesis is controlled by the two-component regulatory system; the membrane-bound sensor histidine kinase and the cytoplasmic response regulator [60,63]. We expect the regulation of Z-geobacillin biosynthesis in strain ZGt-1 to follow what has been described in [60,63]. When the concentration of the secreted lanthipeptide reaches a certain threshold that the membrane-bound sensor histidine kinase (corresponding to the putative ZgeoK in strain ZGt-1) senses, ZgeoK is expected to auto-phosphorylate resulting in the phosphorylation of the cytoplasmic response regulator (corresponding to the putative ZgeoR). The phosphorylated ZgeoR should activate the transcription of the putative *zgeoA* and *zgeoBTC* genes leading to the expression, modification, and transportation of increasing amount of ZgeoA. ZgeoR should also activate the transcription of the genes coding for the immunity proteins, putative ZgeoI and ZgeoGEF, which should protect the producing cell from being inhibited by its own lanthipeptide [60,63]. In general, the immunity protein (corresponding to ZgeoI) is expected to be a peripheral membrane lipoprotein [62] that protects the cell via blocking the pore formation by the secreted lanthipeptide. On the other hand, the ZgeoGEF are putative specialized ABC-transporters. Such ABC-transporters pump the lanthipeptides which have penetrated the membrane back to the exterior environment [60,62]. Based on the roles of immunity proteins as reviewed in [62], the putative gene *zgeoF* is expected to code for the intracellular ATP-binding domain, while the putative *zgeoE* and *zgeoG* are expected to code for the membrane-spanning subunits. In addition to the activation of these genes, based on the biosynthesis scheme [60], we expect ZgeoR to further activate *zgeoR* and *zgeoK* genes. Future experimental research will further clarify the pathway of Z-geobacillin biosynthesis.

• *Geobacillus thermoleovorans* CCB_US3_UF5

*G. thermoleovorans* strain CCB_US3_UF55 is a thermophilic bacterium that was isolated from a hot spring in Malaysia [64]. To our knowledge, the strain has not been experimentally studied for the production of any kind of bacteriocins (Table 2). The antiSMASH analysis of the genome sequence showed that the strain has a putative class-I lanthipeptide cluster coding for one lanthipeptide.

The gene coding for the antiSMASH-predicted lanthipeptide (Table S1) has been inaccurately annotated on the RefSeq genome (Table S2). Here as well, we want to point out that the positions of the coding gene indicated by tblastn analysis did not match the positions reported by antiSMASH 4 and the RefSeq. The reported positions were identical to the positions reported for ‘GTCCBUS3UF5_RS01945’. Although this annotated gene also codes for a lanthipeptide, it is longer than the one that should code for the antiSMASH-predicted lanthipeptide which is 50 aa in length. To resolve this issue, we analyzed the genome of the strain using antiSMASH 3, as we did with *G. kaustophilus* mentioned above. That resulted in the identification of gene positions that agreed with the positions presented by tblastn. InterPro confirmed that the predicted peptide is a lanthipeptide. Moreover, BAGEL4 genome mining tool also retrieved the same lanthipeptide. Therefore, we suggest annotating the lanthipeptide-coding gene on the RefSeq genome at the detected position presented in Table S1.

The antiSMASH-predicted lanthipeptide of *G. thermoleovorans* is identical to lanthipeptide (II) of *G. kaustophilus*. As inferred from BAGEL BLAST, the predicted lanthipeptide has 79% identity to geobacillin I produced by *G. thermodenitrificans* NG80-2 (Table 2; Table S2). Since *G. thermoleovorans* has not been experimentally studied for lanthipeptide production so far, we suggest *G. thermoleovorans* to be a potential class-I lanthipeptide producer, and strain CCB_US3_UF55 as the potential candidate for the species. Noteworthy is that Novotny and Perry, 1992 reported the production of bacteriocins by two strains of *Bacillus thermoleovorans*, the former taxonomic identity of *G. thermoleovorans*, but neither the class nor the aa sequence of the produced bacteriocins were identified [65].

#### 2.3.3. Identification of *Lactococcus*-associated Lanthipeptide Gene Clusters

Members of the genus *Lactococcus* are spherical or ovoid in shape, Gram-positive, facultatively anaerobic, non-motile, and non-spore formers [66]. They belong to the class Bacilli, order Lactobacillales, and family Streptococcaeae [66].

• *Lactococcus lactis*

*L. lactis* is the type species of the genus *Lactococcus* [66]. Strains of this species constitute the majority of lactic acid bacteria in dairy products [67]. They produce different types of bacteriocins, including lanthipeptides [67]. However, not all strains of this species are capable of producing bacteriocins [67]. 

*L. lactis* Strains CV56 and IO-1

Strains CV56 and IO-1 were isolated from different non-dairy sources. Strain CV56 was a clinical isolate [35], while strain IO-1 was isolated from household water [68]. Our analysis indicated that both strains have genes coding for class-I lanthipeptides that are 98% identical, and this is in agreement with the RefSeq annotation (details are given in S.2.3.3.1). Neither of these two strains has been experimentally investigated as a lanthipeptide producer (Table 2).

#### 2.3.4. Identification of *Paenibacillus*-associated Lanthipeptide Gene Clusters

Members of the genus *Panibacillus* are rod-shaped, facultatively anaerobic or strictly aerobic, motile cells [69]. They belong to the class Bacilli, order Bacillales, and family Paenibacillaceae [69].

• *Paenibacillus polymyxa*

*P. polymyxa* is the type species of the genus *Paenibacillus* [69]. It inhabits soils, roots, rhizospheres of crop plants, fermented food, and marine sediments [70]. *P. polymyxa* represents a significant resource of industrially potential products, among which are the antimicrobial agents [70]. Different strains of *P. polymyxa* have been reported as producers of different antimicrobial agents [70,71,72,73,74]. However, production of lanthipeptides by *P. polymyxa* has not been widely studied, except for only a few examples; such as the lanthipeptide produced by *P. polymyxa* strain OSY-DF [74,75], and another produced by strain E681, as discussed below.

*P. polymyxa* CR1

Analysis of the genome sequence of strain CR1 showed that it harbors a class-I lanthipeptide cluster that codes for a lanthipeptide highly similar to paenilan, which was characterized in [36]. The nt sequence of the gene coding for the predicted lanthipeptide (Table S1) is 100% identical to ‘X809_RS07820’, which has been annotated on the RefSeq genome as coding for a hypothetical protein (WP_023987834) (Table S2). The potential of strain CR1 as a class-I lanthipeptide-producer has not been experimentally investigated, but Eastman et al., 2014 mentioned the presence of a lanthipeptide-coding gene on the genome [37]. Interestingly, in the RefSeq genome record of strain CR1, there are genes coding for ‘lantibiotic biosynthesis proteins’; ‘WP_023987835’ and ‘WP_023987837’ positioned downstream of ‘X809_RS07820’. The presence of these neighboring genes thus supports the antiSMASH prediction. Moreover, BAGEL4 genome mining tool as well identified the antiSMASH-predicted lanthipeptide of strain CR1 as a lanthipeptide. These results support our analysis, and suggest that the protein ‘WP_023987834’ annotated on the RefSeq genome of strain CR1 as ‘hypothetical’ is likely to be a lanthipeptide. The identity between paenilan and the predicted lanthipeptide of strain CR1 is 94% (Table 2; Table S2). We noticed that paenilan has not been added to BAGEL databases.

*P. polymyxa* E681

Analysis of the genome sequence of strain E681 showed that it harbors a class-I lanthipeptide cluster. The antiSMASH results are in agreement with the RefSeq annotation and the analysis of Park et al. [36] (details are given in S.2.3.4.1).

*P. polymyxa* strains M1 and SC2

Our analysis indicated that each of strains M1 and SC2 has a class-I lanthipeptide cluster coding for two different lanthipeptides. The aa sequence of lanthipeptide (I) of strain M1 is identical to that of strain SC2. Similarly, the aa sequence of lanthipeptide (II) of strain M1 is identical to that of strain SC2. The presence of a lanthipeptide cluster in strain SC2 was predicted and briefly mentioned by Ma et al., 2011 who sequenced the genome of the strain [38]. However, to our knowledge, and as interpreted from BAGEL databases, neither strain M1 nor strain SC2 has been experimentally investigated as a lanthipeptide-producer so far (Table 2).

The gene coding for the antiSMASH-predicted lanthipeptide (I) of strain M1 (Table S1) has not been annotated on the original genome (Table S2). The gene, however, has been annotated on the RefSeq genome, as discussed below. On the other hand, the gene coding for the predicted lanthipeptide (I) of strain SC2 (Table S1) has not been annotated on the original genome with its full length (Table S2). One part of the predicted gene is overlapping with a gene annotated as coding for subtilin (AKA44201). We want to point out here that the aa sequence of AKA44201 does not match that of the experimentally confirmed ‘lantibiotic subtilin’ (AAB91589; WP_003220055) produced by *B. subtilis*. Accordingly, the antiSMASH-predicted lanthipeptide could not be subtilin. In the RefSeq records of strains M1 and SC2, the antiSMASH-predicted coding genes have been annotated as ‘PPM_RS07455’ and ‘PPSC2_RS36195’, respectively, each of which has been annotated as coding for a hypothetical protein (WP_025676407). Interestingly, on the RefSeq genomes of strains M1 and SC2, there are genes coding for ‘lantibiotic biosynthesis protein’ (WP_013370169) and (WP_014599582), positioned downstream of the predicted lanthipeptide-coding genes. The presence of these neighboring genes supports the antiSMASH prediction. Moreover, BAGEL4 genome mining tool, as well, identified the antiSMASH-predicted lanthipeptide (I) as a lanthipeptide. The potential of strain SC2 as a producer of lanthipeptide (I) was very briefly mentioned by van Heel et al., 2016, but the class of the lanthipeptide was not clarified and only the core sequence of the lanthipeptide was mentioned [30]. Lanthipeptide (I) is 64% identical to paenilan (Table 2).

The gene coding for the antiSMASH-predicted lanthipeptide (II) of strain M1 has not been annotated in the original genome record (Table S2). However, the gene has been annotated on the RefSeq genome, as discussed below. On the other hand, the nt sequence of the gene coding for the predicted lanthipeptide (II) of strain SC2 is 100% identical to ‘PPSC2_07480’ annotated on the original genome as coding for ‘subtilin lantibiotic’ (ADO55541) (Table S2). Similar to what was mentioned above, the predicted lanthipeptide (II) could not be subtilin. On the other hand, in the RefSeq records of both strains, the antiSMASH-predicted genes are 100% identical to ‘PPM_RS07465’ in strain M1 and to ‘PPSC2_RS36205’ in strain SC2. Each of these two genes codes for a hypothetical protein and is positioned within the same genome context of lanthipeptide (I), in each strain (Table S2). BAGEL4 genome mining tool confirmed that the predicted lanthipeptide (II) is a lanthipeptide in both strains. This in turn supports the antiSMASH prediction. Moreover, lanthipeptide (II) is 96% identical to paenilan (Table 2). Taken altogether, these results indicate that the antimicrobial potential of lanthipeptide (I) and (II) should be of interest to explore.

#### 2.3.5. Identification of *Staphylococcus*-associated Lanthipeptide Gene Clusters

Bacterial members of the genus *Staphylococcus* belong to the class Bacilli, order Bacillales, family Staphylococcaceae [76]. They are Gram-positive, cocci, non-motile, and non-spore-forming mesophiles [76]. The genus includes pathogenic and non-pathogenic species [77], and it comprises a number of class-I lanthipeptide-producing strains whose antimicrobial activity has been confirmed, such as Pep 5, epilancin K7, Epicidin 280, and epilancin 15X produced by *S. epidermidis* strains; reviewed in [9], gallidermin produced by *S. gallinarum* DSMZ 4616 [78], and staphyloccin Au-26 [79] and BSA (bacteriocin of *Staphylococcus aureus*) produced by *S. aureus* strains including the methicillin-resistant *S. aureus* (MRSA) strains [8]. Our analysis identified additional *S. aureus* strains harboring putative class-I lanthipeptides.

• *Staphylococcus aureus*

*S. aureus* is the type species of the genus *Staphylococcus* [76]. It is a commensal microorganism, living on the human skin and mucous membranes in the nasal pharynx, but it is also an opportunistic pathogen, causing nosocomial infections associated with high levels of mortality and morbidity [80]. One of the antimicrobially-active lanthipeptides produced by *S. aureus* strains is the BsaA2 type [8].

*S. aureus* 11819-97

The antiSMASH analysis of the genome sequence of strain 11819-97 showed that the strain harbors a class-I lanthipeptide cluster coding for two different lanthipeptides. The antiSMASH results are in agreement with the RefSeq annotation (details are given in S.2.3.5.1). To our knowledge, strain 11819-97 has not been experimentally investigated for the production of lanthipeptides.

*S. aureus* COL

The antiSMASH analysis of the genome sequence of strain COL showed that the strain harbors a class-I lanthipeptide cluster coding for two different lanthipeptides. The antiSMASH results are in agreement with the RefSeq annotation and Daly et al. [8] (details are given in S.2.3.5.2). To our knowledge, strain COL has not been experimentally investigated for the production of lanthipeptides.

*S. aureus* ED133

The antiSMASH analysis of the genome sequence of strain ED133 showed that the strain harbors a class-I lanthipeptide cluster coding for two different lanthipeptides, one of which is identical to BacCH91 produced by *S. aureus* strain CH91 [81]. The antiSMASH results are in agreement with the RefSeq annotations (details are given in S.2.3.5.3). To our knowledge, strain ED133 has not been experimentally investigated for the production of lanthipeptides.

*S. aureus* M1

The antiSMASH analysis of the genome sequence of strain M1 showed that the strain harbors a class-I lanthipeptide cluster coding for two different lanthipeptides. The antiSMASH results are in agreement with the RefSeq annotation (details are given in S.2.3.5.4). To our knowledge, strain M1 has not been experimentally investigated for the production of lanthipeptides.

*S. aureus* MSSA476

The antiSMASH analysis of the genome sequence of strain MSSA476 showed that the strain MSSA476 harbors a class-I lanthipeptide cluster coding for two different lanthipeptides. The antiSMASH results are in agreement with the RefSeq annotation and Daly et al. [8] (details are given in S.2.3.5.5). To our knowledge, strain MSSA476 has not been experimentally investigated for the production of lanthipeptides (Table 2).

*S. aureus* MW2

The antiSMASH analysis of the genome sequence of strain MW2 showed that the strain harbors a class-I lanthipeptide cluster coding for two different lanthipeptides. The antiSMASH results are in agreement with the RefSeq annotation and Daly et al. [8] (details are given in S.2.3.5.6). To our knowledge, strain MW2 has not been experimentally investigated for the production of lanthipeptides.

*S. aureus* NCTC 8325

The antiSMASH analysis of the genome sequence of strain NCTC 8325 showed that the strain harbors a class-I lanthipeptide cluster coding for two different lanthipeptides. The antiSMASH results of lanthipeptide (I) are in agreement with the RefSeq annotation and Daly et al. [8] (details are given in S.2.3.5.7).

Analysis of the aa sequence of the antiSMASH-predicted lanthipeptide (II) (Table S1) showed that the predicted gene has neither been annotated in the original nor in the RefSeq genome records (Table S2). However, analyzing the aa sequence of the predicted peptide using InterPro confirmed that the peptide was properly predicted by antiSMASH as a lanthipeptide. Moreover, BAGEL4 genome mining tool also confirmed that the predicted peptide is a lanthipeptide. However, the position of the lanthipeptide-coding gene predicted by antiSMASH was incorrect with one nt difference. This was also confirmed by tblastn analysis; therefore, we corrected the position of the predicted gene (Table S1). It is noteworthy that antiSMASH 3, however, did not predict this lanthipeptide. BAGEL BLAST showed that lanthipeptide (II) is 83% identical to BsaA2 (Table 2). Taken altogether, we recommend annotating the lanthipeptide-coding gene in the RefSeq genome record of the strain on the position presented in Table S1. This predicted lanthipeptide has not been reported previously (Table 1).

*S. aureus* RF122

The antiSMASH analysis of the genome sequence of strain RF122 (also known as ET3-1) showed that the strain harbors a class-I lanthipeptide cluster coding for two different lanthipeptides. The antiSMASH results are in agreement with the RefSeq annotations (details are given in S.2.3.5.8).

*S. aureus* T0131

The antiSMASH analysis of the genome sequence of strain T0131 showed that the strain harbors a class-I lanthipeptide cluster coding for two different lanthipeptides. The antiSMASH results are in agreement with the RefSeq annotation (details are given in S.2.3.5.9). To our knowledge, strain T0131 has not been reported as a producer of lanthipeptides.

*S. aureus* strains USA300 FPR3757 and USA300_TCH1516

Analysis of the genome sequences of strains USA300 FPR3757 and USA300_TCH1516 using antiSMASH showed that each of the strains harbors a class-I lanthipeptide cluster coding for two different lanthipeptides. The antiSMASH results of both strains are in agreement with the RefSeq annotations and Daly et al. [8] (details are given in S.2.3.5.8.10). To our knowledge, neither of the strains has been experimentally investigated for the production of lanthipeptides.

*S. aureus* Z172

The antiSMASH analysis of the genome sequence of strain Z172 showed that the strain harbors a class-I lanthipeptide cluster coding for two different lanthipeptides. The antiSMASH results are in agreement with the RefSeq annotation (details are given in S.2.3.5.8.11). To our knowledge, strain Z172 has not been reported for its lanthipeptide production.

Other strains of *S. aureus*

In addition to the above mentioned lanthipeptides coded on the genomes of different *S. aureus* strains, antiSMASH analysis indicated the potential of additional *S. aureus* strains as lanthipeptide producers. Strains Bmb 9393, Newman, TW20, VC40, and LGA251 have two lanthipeptides coded in the same cluster. These strains have not been experimentally investigated (Table 2). The antiSMASH results are in agreement with the RefSeq annotations (details are given in S.2.3.5.8).

#### 2.3.6. Identification of *Streptococcus*-associated Lanthipeptide Gene Clusters

Bacterial members of the genus *Streptococcus* belong to the class Bacilli, order Lactobacillales, family Streptococcaceae [82]. They are Gram-positive, spherical or ovoid in shape, occur in chains or pairs, non-motile, and non-spore-formers [82]. Most of the species are facultatively anaerobic [82]. While some *Streptococcus* species are part of the human microflora, others are pathogenic [83].

Streptococci are known bacteriocin-producers, but only a few species, mainly pathogenic, have been in the focus of bacteriocin-isolation and characterization [84]. Most of the produced bacteriocins are antimicrobially-active lanthipeptides [84], among which are the class-I lanthipeptides streptin [85], nisin U [86], and some mutacins [87,88,89]. Marsh et al., 2010, identified putative class-I lanthipeptides in some streptococci [15]. In the current study, we have identified putative class-I lanthipeptides in species and strains that have not previously been described as potential lanthipeptide producers.

• *Streptococcus intermedius*

*S. intermedius* is usually a commensal microorganism of the mouth, and the upper respiratory and intestinal tracts, but it can cause diseases in immunocompromised individuals [90]. To our knowledge, none of the members of *S. intermedius* species has been reported as a lanthipeptide producer previously. This in turn makes investigating strains of *S. intermedius* as lanthipeptide producers of significant interest. In the current study, we are presenting two strains; B196 and C270 as putative class-I lanthipeptide producers (Table 2).

*S. intermedius* B196

*S. intermedius* strain B196 is a clinical isolate that was obtained from a patient with broncho-pulmonary disease and a combination of joint, muscle, and bone infections [91].

The antiSMASH analysis of the genome sequence of strain B196 showed that the strain has a putative class-I lanthipeptide cluster. Analysis of the aa sequence of the antiSMASH-predicted lanthipeptide (Table S1) indicated that the predicted coding gene has not been annotated on the original genome (Table S2). However, the gene has been annotated on the RefSeq as ‘SIR_RS12740’ which codes for a hypothetical protein (WP_037582862) (Table S2). We checked the neighboring genes in the RefSeq record and found that the ‘biosynthesis protein’ (WP_021002593), coded downstream of the putative lanthipeptide, has a lanthionine synthetase C-like domain (IPR007822) as indicated by InterPro analysis. Therefore, this ‘biosynthesis protein’ is most likely to be LanC. Moreover, we found that the ‘hypothetical protein’ (WP_041787401), coded upstream of the putative lanthipeptide, has “lantibiotic dehydratase, N-terminal” (Lant_dehydr_N) and “lantibiotic dehydratase, C-terminal” (Lant_dehydr_C) domains which characterize LanB proteins. Therefore, the ‘hypothetical protein’ (WP_041787401) is most likely to be LanB. The presence of genes coding for these two lanthipeptide-modifying enzymes makes it likely that the protein (WP_037582862) annotated on the RefSeq as a ‘hypothetical protein’ and predicted as a lanthipeptide by antiSMASH (Table S1) is a lanthipeptide. Moreover, BAGEL4 genome mining tool as well identified the existence of a class-I lanthipeptide cluster and confirmed the presence of LanB and LanC. Therefore, we suggest re-annotating these proteins in the RefSeq record. BAGEL BLAST showed that the predicted lanthipeptide did not have any hits to any known small bacteriocins (Table 2). These results indicate that the antimicrobial potential of lanthipeptide (I) and (II) should be of significant interest to explore.

*S. intermedius* C270

*S. intermedius* strain C270 is a clinical isolate that was obtained from the respiratory tract of a patient with a broncho-pulmonary disease [91]. The antiSMASH analysis of the genome sequence of strain C270 showed that the strain has a putative class-I lanthipeptide cluster. The antiSMASH results are in agreement with the RefSeq annotation (details are given in S.2.3.6.1).

• *Streptococcus pasteurianus*

*S. pasteurianus* is also known as *S. gallolyticus* subsp. *pasteurianus*. Members of this species are part of the intestinal microflora in humans but can also cause diseases especially in immunocompromised patients [92]. Zhang et al., 2012 briefly mentioned the potential of this species as a class-I lanthipeptide producer [93]. To our knowledge, members of *S. pasteurianus* have not been experimentally investigated as lanthipeptide producers. In the current study, we are presenting *S. pasteurianus* strain ATCC43144 as a potential class-I lanthipeptide producer.

*S. pasteurianus* Strain ATCC 43144

Strain ATCC 43144 is part of the normal flora of the gut in human, but it also causes various diseases [39]. *In silico* analysis of its genome sequence, carried out by Lin et al., 2011, indicated that the strain acquired a biosynthetic gene cluster including a lanthipeptide-coding gene, nisin U, from another *Streptococcus* strain [39]. Our analysis using antiSMASH confirmed the presence of a class-I lanthipeptide cluster. The antiSMASH results are in agreement with the RefSeq annotation (details are given in S.2.3.6.2).

• *Streptococcus pyogenes*

*S. pyogenes* is the type species of the genus *Streptococcus* [82]. *S. pyogenes* strains are bacteriocin-producers. Streptin is a known class-I lanthipeptide that has antibacterial activity produced by different strains of *S. pyogenes* [84].

*S. pyogenes* strain MGAS9429

Strain MGAS9429 has not been previously reported as a lanthipeptide producer. Our analysis indicated that this *S. pyogenes* strain as well is a putative streptin-producer. The antiSMASH results are in agreement with the RefSeq annotation (details are given in S.2.3.6.3).

Other strains of *S. pyogenes*

Analysis using antiSMASH indicated that strains MGAS6180, MGAS10270, and MGAS10750 of *S. pyogenes* harbor class-I lanthipeptide clusters. The antiSMASH results are in agreement with the RefSeq annotations and with the results of Marsh et al., 2010 [15] (details are given in S.2.3.6.4).

• *Streptococcus suis*

*S. suis* is a swine pathogen that can be transmitted to humans; it causes meningitis and septic shock syndrome that may lead to death [94]. Some strains of this species have been reported as lanthipeptide producers [95,96].

*S. suis* strains JS14 and SC070731

The antiSMASH analysis of the genome sequences of strains JS14 and SC07073 showed that each of the strains has a putative class-I lanthipeptide cluster (Table S1). The lanthipeptide production potential of strains JS14 and SC07073 has not yet been investigated. The antiSMASH results are in agreement with the RefSeq annotation (details are given in S.2.3.6.5).

## 3. Methods

### 3.1. Selection of the Genome Sequences to be Analyzed

All 252 available firmicute RefSeq genome sequences were downloaded from the bacterial genome section of NCBI (ftp://ftp.ncbi.nlm.nih.gov/genomes/Bacteria) on 28 April 2015. They were then subjected to different analyses. Due to the long time period of analysis, a check was made before finalizing the study to see if any additional firmicute species had been added to the assembly section of NCBI (https://www.ncbi.nlm.nih.gov/assembly). Only complete genomes belonging to the RefSeq category ‘Representative’ were chosen (20 June 2018). Out of the 292 genomes, only four; *Staphylococcus capitis* subsp. *capitis* strain AYP1020, *Bacillus mycoides* strain ATCC 6462, *Streptococcus salivarius* strain NCTC 8618, and *Paenibacillus* sp. BD3526 contained genes coding for class-I lanthipeptides that we had not analyzed before. These species have not been included in the detailed analysis.

### 3.2. Identification of Class-I Lanthipeptides Using antiSMASH

For the identification of class-I lanthipeptide-coding genes, all genome sequences were subjected to antiSMASH analysis. The antiSMASH standalone docker version (4.0.2) was run on GenBank genome records [23]. For certain genome sequences, where the gene position reported by antiSMASH 4.0.2 did not match that reported by tblastn, the antiSMASH standalone for Ubuntu (3.0.4) [24] was run on genome fasta files.

Using custom made scripts, class-I lanthipeptides including their leader peptides were extracted from the antiSMASH (4.0.2) output files with extension final.gbk. The aa residues in the leader peptide segment of the full peptide were converted to lowercase. The sequences were aligned using MUSCLE (version 3.8.31) with default settings [97]. The resulting alignment was then calibrated using the refine option. The output from antiSMASH, both version 3.0.4 and 4.0.2, together with the source code for in-house developed programs and a description of how the analysis was conducted are found at: http://130.235.46.10/Lanthipeptides (last checked on the 5 September 2018).

A set of previously described lanthipeptides that support the predictions of antiSMASH is presented in (Table S6).

### 3.3. Analysis of the Identified Class-I Lanthipeptides

#### 3.3.1. NCBI BLAST Analysis

The antiSMASH-predicted lanthipeptides were analyzed with various NCBI BLAST programs (2.8.0+) [25] using non-redundant and RefSeq as databases, where gene annotation and position of the lanthipeptide-coding gene were retrieved. Additionally, manual inspections of genome files—the original genome records and the RefSeq records—were performed.

#### 3.3.2. BAGEL Analysis

Each of the antiSMASH-predicted lanthipeptides was subjected to BAGEL4 BLAST analysis against class-I and class-II bacteriocin databases [26]. Moreover, we referred to BAGEL4 databases to check whether the strain, which was predicted by antiSMASH to harbor a class-I lanthipeptide biosynthesis gene cluster(s), has been reported as a lanthipeptide-producer, or yet to be. Additionally, for certain genome sequences given in the main text, BAGEL4 genome mining tool [26] was employed as well in addition to antiSMASH in order to identify class-I lanthipeptides and confirm the antiSMASH predictions.

#### 3.3.3. InterPro Analysis

The protein family of a given lanthipeptide was detected using the InterProScan sequence search tool [98]. It was used for certain lanthipeptides, given in the main text, to confirm that the prediction of the lanthipeptide by antiSMASH 3 and antiSMASH 4 was correct.

### 3.4. Mapping of Z-Geobacillin Biosynthetic Pathway

The putative biosynthetic pathway of Z-geobacillin in *Geobacillus* sp. ZGt-1 was plotted using KAAS [61].

### 3.5. Alignment of Lanthipeptide Sequences of Geobacillus strains

The three lanthipeptide sequences of *G. kaustophilus* HTA426, *Geobacillus* sp. ZGt-1 (Z-geobacillin), and *G. thermodenitrificans* NG80-2 (geobacillin I) were aligned using the ClustalW Multiple alignment option in BioEdit 7.0.5.3 [58].

## 4. Conclusions

We identified 69 class-I lanthipeptides out of the analyzed 252 firmicute completely-sequenced genomes. Out of these 69 lanthipeptides, we have presented a list of seven putative novel class-I lanthipeptide candidates (Table 1). In addition, we identified one more lanthipeptide; Z-geobacillin, which we inspected closely (Table 1). We also presented a list of 40 bacterial strains that have not been investigated so far as class-I lanthipeptide producers. We proposed these strains as good nominees for experimental investigations of their lanthipeptide production potential (Table 2). The presented *in silico* genome analysis-based strategy for the identification of lanthipeptides was conducted by employing the antiSMASH software and BAGEL databases, and BAGEL genome mining tool in some cases, in order to screen for class-I lanthipeptide biosynthetic gene clusters, and identify putative lanthipeptides. The presented proposal is meant to benefit researchers interested in producing lanthipeptides *in vitro*. It reduces the time required for finding and prioritizing the ‘right’ candidates to be investigated. As suggested by Xin et al., 2015, even lanthipeptides that show similarity to known ones can still constitute potentially novel lanthipeptides, especially when some studies have demonstrated that lanthipeptide engineering can lead to the production of lanthipeptides with improved characteristics, such as thermal stability and broader spectrum of inhibitory actions [31]. In addition to proposing putative lanthipeptides and potential strains, we identified lanthipeptide-coding genes that have been overlooked during the annotation of some genome sequences (Table 1).

Besides the *in silico* analysis-based strategy, we presented an example on a combinatorial strategy. This strategy starts with the conventional (culture-based) approach and is complemented with the *in silico* analysis. The conventional approach for cultivable bacterial strains launches with sampling from preferably special ecosystems; such as extreme habitats [19], cultivation, and isolation of microorganisms, screening for their antimicrobial activity using specific biological assays, then proceeding with *in silico* analysis of the genomic data of the isolated strain for identifying the potential antimicrobial compound(s).

As a potential nominee, we presented the thermophilic *Geobacillus* sp. strain ZGt-1, which was isolated from Zara hot spring in Jordan and the genome was sequenced [21,22], as a putative producer of the lanthipeptide, Z-geobacillin. This lanthipeptide will be purified and characterized, and its antimicrobial activity will be evaluated in future studies.

Each strategy has its own pros and cons; the *in silico* analysis-based strategy proved its success [1]. It is an efficient avenue since it shortens the time and efforts spent on screening for potential producers compared to the wet-lab screening [14]. It is also independent of the bias which the selected laboratory conditions could have on the lanthipeptide production during cultivating the putative producer [14,99]. Therefore, the *in silico* analysis minimizes the probability of overlooking producing strains whose lanthipeptide production might not be expressed under the selected cultivation conditions *in vitro* [14]. It is noteworthy here that the *in silico* predictions may vary depending on the employed prediction methodology as shown by Walsh et al., 2015 [99]. In our study, we found that the analyzed bacterial strains that have been previously described as class-I lanthipeptide producers were successfully predicted by antiSMASH (Table S6). Concerning the proved lanthipeptides that have not yet been listed in BAGEL databases, we will notify the team of programmers of the BAGEL software to add those lanthipeptides to the database.

The *in silico* analysis, however, does not guarantee that the predicted lanthipeptide will be expressed *in vitro*, and does not confirm its antimicrobial activity [1]. Therefore, the *in silico* analysis-based strategy needs to be pursued with experimental validation to ensure the production of the lanthipeptide and evaluate its antagonistic activity against different indicator microorganisms of interest [99].

On the other hand, the conventional approach is time-consuming and laborious [14] but selecting a unique ecological niche for isolating potential microorganisms raises the probability of discovering novel natural compounds.

Combining the conventional approach with the *in silico* analysis is an effective route towards unraveling novel lanthipeptides. Sequencing and analyzing the genome of the isolated bacterium, using the right genome analysis tools, provides valuable insights into the genes coding for secondary metabolites. Accordingly, identification of the lanthipeptide-coding genes among all the other genes coding for metabolites produced by the strain in question becomes accessible. Successively, genome sequencing and analysis aid in designing the right methodology for the *in vitro* production and purification of the lanthipeptide of interest.

## Figures and Tables

**Table 1:** Bacterial genomes where a total of eight putative novel class-I lanthipeptides were identified. Annotations of the lanthipeptide-coding gene in the original genome and the RefSeq records are presented.

Bacterial Species, Strain (Lanthipeptide Reference Number)	RefSeq Genome Accession Number	Annotation of the Lanthipeptide-Coding Gene	
		Original Genome Record	RefSeq Genome Record
*Bacillus thuringiensis* serovar finitimus YBT-020	NC_017200	‘Hypothetical protein’	Unannotated
*Geobacillus* sp. ZGt-1	LDPD01000000 ^1^	Lanthipeptide	Unavailable in RefSeq
*Paenibacillus polymyxa* M1 (I)	NC_017542	Unannotated	‘Hypothetical protein’
*Paenibacillus polymyxa* M1 (II)	NC_017542	Unannotated	‘Hypothetical protein’
*Paenibacillus polymyxa* SC2 (I) ^2^	NC_014622	Partly inaccurately annotated	‘Hypothetical protein’
*Paenibacillus polymyxa* SC2 (II)	NC_014622	Incorrectly annotated as coding for subtilin	‘Hypothetical protein’
*Staphylococcus aureus* NCTC 8325 (II)	NC_007795	Unannotated	Unannotated
*Streptococcus intermedius* B196 ^3^	NC_022246	Unannotated	‘Hypothetical protein’

^1^ The presented genome sequence accession number for *Geobacillus* sp. ZGt-1 belongs to the original genome record; the whole genome shotgun (WGS) project. ^2^ Only the core sequence of this lanthipeptide was briefly mentioned in another study, but the lanthipeptide was not clearly classified as class-I (details are given in the text). ^3^ *S. intermedius* has not been reported in literature as a class-I lanthipeptide-producer.

**Table 2:** Bacterial strains with predicted lanthipeptide production potential, representing possible subjects for future experimental investigations. Strains typed in bold belong to a firmicute species that has not been reported in literature as a producer of class-I lanthipeptides, but the lanthipeptide production potential of its presented strains has been addressed in the current study.

Bacterial Strain	RefSeq Genome Accession Number	Total Number of Harbored Class-I Lanthipeptides	Lanthipeptide Reference Number ^1^	Identity to Experimentally Verified Lanthipeptide ^2^	Reference ^3^
*Bacillus clausii* KSM-K16	NC_006582	1	- ^4^	56% clausin	[30], and this study
*Bacillus megaterium* QM B1551	NC_014023	2	I and II	56% gallidermin	[30,31], and this study
*Bacillus subtilis* BSn5	NC_014976	1	- ^4^	100% subtilomycin	[32,33], and this study
*Bacillus subtilis spizizenii* W23	NC_014479	1	- ^4^	100% subtilin	This study
*Bacillus thuringiensis* serovar finitimus YBT-020	NC_017200	1	- ^4^	No hits	This study
*Bacillus thuringiensis* serovar IS5056	NC_020394	5	I and II III IV V	100% thuricin 4A-4 86% thuricin 4A-4 84% thuricin 4A-4 82% thuricin 4A-4	[31], and this study
*Bacillus thuringiensis* YBT 1518	NC_022873	2	I II	53% gallidermin 51% gallidermin	This study
*Geobacillus kaustophilus* HTA426	NC_006510	2	I II	91% geobacillin I 79% geobacillin I	[15,34], and this study
*Geobacillus* sp. ZGt-1	LDPD01000000	1	- ^4^	91% geobacillin I	This study
*Geobacillus thermoleovorans* CCB_US3_UF5	NC_016593	1	- ^4^	79% geobacillin I	This study
*Lactococcus lactis* CV56	NC_017486	1	- ^4^	100% nisin A	[15,35], and this study
*Lactococcus lactis* IO-1	NC_020450	1	- ^4^	100% nisin Z	This study
*Paenibacillus polymyxa* CR1	NC_023037	1	- ^4^	94% paenilan	[36,37], and this study
*Paenibacillus polymyxa* M1	NC_017542	2	I II	64% paenilan 96% paenilan	[36], and this study
*Paenibacillus polymyxa* SC2	NC_014622	2	I II	64% paenilan 96% paenilan	[30,36,38], and this study
*Staphylococcus aureus* 11819-97	NC_017351	2	I II	100% BsaA2 83% BsaA2	This study
*Staphylococcus aureus* Bmb 9393	NC_021670	2	I II	100% BsaA2 83% BsaA2	This study
*Staphylococcus aureus* COL	NC_002951	2	I II	100% BsaA2 79% BsaA2	[8], and this study
*Staphylococcus aureus* ED133	NC_017337	2	I II	100% BacCH91 85% BsaA2	[30], and this study
*Staphylococcus aureus* LGA251	NC_017349	2	I II	81% BsaA2 85% BsaA2	This study
*Staphylococcus aureus* M1	NC_021059	2	I II	100% BsaA2 83% BsaA2	This study
*Staphylococcus aureus* MSSA476	NC_002953	2	I II	100% BsaA2 83% BsaA2	[8], and this study
*Staphylococcus aureus* MW2	NC_003923	2	I II	100% BsaA2 83% BsaA2	[8], and this study
*Staphylococcus aureus* NCTC 8325	NC_007795	2	I II	100% BsaA2 83% BsaA2	[8], and this study
*Staphylococcus aureus* Newman	NC_009641	2	I II	100% BsaA2 83% BsaA2	[8], and this study
*Staphylococcus aureus* T0131	NC_017347	2	I II	100% BsaA2 83% BsaA2	This study
*Staphylococcus aureus* TW20	NC_017331	2	I II	100% BsaA2 83% BsaA2	This study
*Staphylococcus aureus* USA300 FPR3757	NC_007793	2	I II	100% BsaA2 83% BsaA2	[8], and this study
*Staphylococcus aureus* USA300_TCH1516	NC_010079	2	I II	100% BsaA2 83% BsaA2	[8], and this study
*Staphylococcus aureus* VC40	NC_016912	2	I II	100% BsaA2 83% BsaA2	This study
*Staphylococcus aureus* Z172	NC_022604	2	I II	100% BsaA2 83% BsaA2	This study
***Streptococcus intermedius* B196**	NC_022246	1	- ^4^	No hits	This study
***Streptococcus intermedius* C270**	NC_022237	1	- ^4^	81% nisin F	This study
*Streptococcus pasteurianus* ATCC 43144	NC_015600	1	- ^4^	91% nisin U	[39], and this study
*Streptococcus pyogenes* MGAS6180	NC_007296	1	- ^4^	100% streptin	[15], and this study
*Streptococcus pyogenes* MGAS9429	NC_008021	1	- ^4^	100% streptin	This study
*Streptococcus pyogenes* MGAS10270	NC_008022	1	- ^4^	100% streptin	[15], and this study
*Streptococcus pyogenes* MGAS10750	NC_008024	1	- ^4^	98% streptin	[15], and this study
*Streptococcus suis* JS14	NC_017618	1	- ^4^	100% suicin 90-1330	This study
*Streptococcus suis* SC070731	NC_020526	1	- ^4^	100% suicin 90-1330	This study

**^1^** The amino acid (aa) sequences of the lanthipeptides are presented in Table S1. ^2^ Identity (%) to the experimentally verified lanthipeptides is based on the bacteriocin genome mining tool (BAGEL) BLAST, the literature, or both. ^3^ The cited references represent studies where the respective strain was mentioned as a potential lanthipeptide producer based on an *in silico* analysis. In some of these studies, the lanthipeptide aa sequences have not been determined. ^4^ (-) Indicates the lack of a lanthipeptide reference number because the strain has only one class-I lanthipeptide.

## Supplementary Materials

Supplementary materials can be found at http://www.mdpi.com/1422-0067/19/9/2650/s1.

## Abbreviations

aa	amino acid
ABC transporters	ATP-binding cassette transporters
antiSMASH	Antibiotics and Secondary Metabolite Analysis Shell
BAGEL	bacteriocin genome mining tool
BSA	bacteriocin of *Staphylococcus aureus*
Dha	dehydroalanine
Dhb	dehydrobutyrine
GeoAI	geobacillin I precursor peptide
GeoB	geobacillin I dehydratase enzyme
GeoC	geobacillin I cyclase enzyme
GeoGEF	self-immunity ABC transporter proteins
GeoI	geobacillin I self-immunity protein
GeoK	geobacillin I sensor histidine kinase protein; part of the two-component response regulatory system
GeoR	geobacillin I response regulatory protein; part of the two-component response regulatory system
GeoTI	geobacillin I ABC transporter protein
KAAS	KEGG Automatic Annotation Server
KEGG	Kyoto Encyclopedia of Genes and Genomes
Lan	lanthionine
LanP	lanthipepttide processing protease
MeLan	(2*S*,3*S*,6*R*)-3-methyllanthionine
Nt	nucleotide
PGAP	prokaryotic genome annotation pipeline
RODEO	Rapid ORF Description and Evaluation Online genome-mining platform
ZgeoA	Z-geobacillin precursor peptide
ZgeoB	Z-geobacillin dehydratase enzyme
ZgeoC	Z-geobacillin cyclase enzyme
ZgeoGEF	Z-geobacillin self-immunity ABC transporter proteins
ZgeoI	Z-geobacillin self-immunity protein
ZgeoK	Z-geobacillin sensor histidine kinase protein; part of the two-component response regulatory system
ZgeoR	Z-geobacillin response regulatory protein; part of the two-component response regulatory system
ZgeoT	Z-geobacillin ABC transporter protein
