# Supplementary material for: Identification of Putative Novel Class-I Lanthipeptides in Firmicutes: A Combinatorial In Silico Analysis Approach Performed on Genome Sequenced Bacteria and a Close Inspection of Z-Geobacillin Lanthipeptide Biosynthesis Gene Cluster of the Thermophilic Geobacillus sp. Strain ZGt-1
Source: Int J Mol Sci. 2018 Sep 6;19(9):2650. doi: 10.3390/ijms19092650 (PMC6165006; doi:10.3390/ijms19092650)
Supplement: Supplementary file 1 [file ijms-19-02650-s001.pdf]

Electronic Supplementary Materials

Identification of Putative Novel Class-I Lanthipeptides in Firmicutes: A Combinatorial *In Silico* Analysis Approach Performed on Genome Sequenced Bacteria and a Close Inspection of Z-geobacillin Lanthipeptide Biosynthesis Gene Cluster of the Thermophilic *Geobacillus* sp. Strain ZGt-1

Rawana N. Alkhalili [1,*] and Björn Canbäck [2]

[1] Biotechnology, Department of Chemistry, Lund University Lund, SE-221 00, Sweden
[2] Department of Biology, Lund University, Lund SE-221 00, Sweden; Bjorn.Canback@biol.lu.se
* Correspondence: Rawana.Alkhalili@biotek.lu.se

Table S.1. Amino acid sequences of the unmodified antiSMASH-detected lanthipeptides and the corresponding nucleotide sequences. Sequences in lower case correspond to the leader peptide. The presented gene positions are based on those predicted by antiSMASH and confirmed by BLAST analysis. When the gene start position value is larger than that of the end position, the gene is located on the reverse strand. Entries typed in bold represent data related to the putative novel lanthipeptides.

**Bacterial species, strain (lanthipeptide reference number), unmodified aa sequence of lanthipeptide**	**Bacterial species, strain (lanthipeptide reference number), lanthipeptide nucleotide sequence**	**RefSeq genome accession number**	**Start position**	**End position**
>*Bacillus clausii* KSM-K16<br>mekafdldlevvhtkakdvqpdFTSVSFCTPGCGETGS<br>FNSFCC	> *Bacillus clausii* KSM-K16<br>ATGGAAAAAGCTTTTGACTTGGATTTGGAA<br>GTAGTACACACGAAAGCGAAGGATGTACA<br>ACCAGATTTCACAAGCGTCAGTTTCTGTAC<br>TCCTGGTTGCGGGGAAACGGGTAGCTTTAA<br>TAGCTTCTGCTGCTAA	NC_006582	3706418	3706284
> *Bacillus megaterium* QM B1551 (I)<br>mekafdldlevvhtkakdvqpdFTSVSFCTPGCGETGS<br>FNSFCC	>*Bacillus megaterium* QM B1551 (I)<br>ATGAATAACGTAAAAAACCTTTTTGATTTA<br>GATGTACAAGTAACAACTGCTTCAAGTGAT<br>GTAGATCCACAAATTACAAGTGTATCTTTA<br>TGTACACCAGGTTGTGGAGATACAGGTAGT<br>TGGAACAGCTTCTGCTGCTAA	NC_014023	137678	137538
>*Bacillus megaterium* QM B1551 (II)<br>mnnvknlfdldvqvttassdvdpqITSVSLCTPGCGDT<br>GSWNSFCC	>*Bacillus megaterium* QM B1551 (II)<br>ATGAATAATGTAAAAAACCTTTTTGATTTA<br>GATGTACAAGTAACAACTGCTTCAAGTGAT<br>GTAGATCCACAAATTACAAGTGTATCTTTA<br>TGTACACCAGGTTGTGGAGATACAGGTAGC<br>TGGAACAGCTTCTGCTGTTAA	NC_014023	137893	137753
>*Bacillus subtilis* BSn5<br>meknnifdldinkkmestsevsaqTWATIGKTIVQSVK<br>KCRTFTCGCSLGSCSNCN	>*Bacillus subtilis* BSn5<br>ATGGAGAAGAATAATATTTTTGACCTAGAC<br>ATTAATAAAAAGATGGAATCTACTAGTGA<br>GGTTTCTGCTCAAACTTGGGCTACAATCGG<br>AAAAACTATTGTGCAGTCTGTGAAAAAAT<br>GTAGAACATTTACTTGTGGATGTTCGCTAG<br>GCTCTTGCAGTAACTGTAACTAA	NC_014976	2428804	2428974
>*Bacillus subtilis spizizenii* TU-B-10<br>mskfddfdldvvkvskqdskitpqWKSESVCTPGCVT<br>GLLQTCFLQTITCNCKISK	>*Bacillus subtilis spizizenii* TU-B-10<br>ATGTCAAAGTTCGATGATTTCGATTTGGAT<br>GTTGTGAAAGTCTCTAAGCAAGACTCAAA<br>AATCACTCCTCAATGGAAAAGTGAGTCAGT<br>TTGTACACCAGGGTGTGTAACTGGTCTATT<br>GCAAACTTGCTTCCTTCAAACAATCACTTG<br>TAACTGCAAAATCTCTAAATAA	NC_016047	3414730	3414560
>*Bacillus subtilis spizizenii* W23<br>mskfddfdldvvkvskqdskitpqWKSESLCTPGCVT<br>GALQTCFLQTLTCNCKISK	>*Bacillus subtilis spizizenii* W23<br>ATGTCAAAGTTCGATGATTTCGATTTGGAT<br>GTTGTGAAAGTCTCTAAACAAGACTCAAA<br>AATCACTCCGCAATGGAAAAGTGAATCAC<br>TTTGTACACCAGGATGTGTAACTGGTGCAT<br>TGCAAACTTGCTTCCTTCAAACACTAACTT<br>GTAACTGCAAAATCTCTAAATAA	NC_014479	3256972	3256802
**> *Bacillus thuringiensis* serovar finitimus YBT-020<br>mdnksqfedfdlnlyeeidysedgsvqsySSWGGTNS<br>STSIACSLLTNVTEGCNWC**	**>*Bacillus thuringiensis*_serovar finitimus YBT-020<br>ATGGACAACAAATCGCAATTTGAAGATT<br>TTGATTTAAACTTATATGAAGAAATTGAT<br>TATAGTGAAGATGGATCAGTGCAATCAT<br>ACTCATCTTGGGGGGGAACGAATAGTTC<br>TACATCAATTGCATGTTCTTTATTAACTA<br>ATGTTACTGAAGGATGTAATTGGTGCTA<br>A**	**NC_017200**	**1205213 [1]**	**1205383 [1]**

>*Bacillus thuringiensis* serovar IS5056 (I)<br>mnkelfdldinkkmetptemtaqTWTTIVKVSKAVCKTGTCICTTSCSNCK	>*Bacillus thuringiensis* serovar IS5056 (I)<br>ATGAATAAAGAACTATTTGATTTAGATATTAATAAGAAAATGGAAACACCTACTGAAATGACAGCACAAACTTGGACAACTATTGTTAAAGTCTCAAAGGCTGTTTGTAAAACAGGAACTTGTATTTGTACTACTTCATGCTCTAATTGTAAGTAA	NC_020394	132195	132040
>*Bacillus thuringiensis* serovar IS5056 (II)<br>mnkelfdldinkkmetptemtaqTWTTIVKVSKAVCKTGTCICTTSCSNCK	>*Bacillusthuringiensis* serovar IS5056 (II)<br>ATGAATAAAGAACTATTTGATTTAGATATTAATAAGAAAATGGAAACACCTACTGAAATGACAGCACAAACTTGGACAACTATTGTTAAAGTCTCAAAGGCTGTTTGTAAAACAGGAACTTGTATTTGTACTACTTCATGCTCTAATTGTAAGTAA	NC_020394	132712	132557
>*Bacillus thuringiensis* serovar IS5056 (III)<br>mdkelfdldinkkmetptemtaqTWTSILKVSRAPCKSGTCLCTTSCSNCK	>*Bacillus thuringiensis* serovar IS5056 (III)<br>ATGGATAAAGAACTATTTGATTTAGATATTAATAAGAAAATGGAAACACCTACTGAAATGACAGCACAAACTTGGACTAGTATTTTAAAGGTGTCAAGGGCTCCTTGTAAATCAGGAACTTGTCTTTGTACTACTTCATGCTCTAATTGTAAATAA	NC_020394	133239	133084
>*Bacillus thuringiensis* serovar IS5056 (IV)<br>mnkelfdldinkkmeaptemtvqtwGTVVKVSKAICKTGTCIGTISCTNCK	>*Bacillus thuringiensis* serovar IS5056 (IV)<br>ATGAATAAAGAACTATTTGATTTAGATATTAATAAGAAAATGGAAGCACCTACTGAAATGACTGTACAAACTTGGGGAACGGTTGTTAAAGTATCAAAGGCTATTTGTAAAACAGGAACTTGTATTGGCACTATTTCATGCACTAATTGTAAGTAA	NC_020394	133674	133519
>*Bacillus thuringiensis* serovar IS5056 (V)<br>mnkelfdldinkkmetptemtaqtvPTTIFVSRSVCKTLTCICTISCSNCK	>*Bacillus thuringiensis* serovar IS5056 (V)<br>ATGAATAAAGAATTATTTGATTTAGATATTAATAAGAAAATGGAAACACCTACTGAAATGACTGCACAAACTGTGCCAACAACTATTTTTGTATCAAGAAGTGTTTGTAAAACATTAACTTGTATTTGTACTATTTCATGCTCTAATTGTAAGTAA	NC_020394	134225	134070
>*Bacillus thuringiensis* YBT 1518 (I)<br>mineknlfdldvqvttatgdvdpqITSISACTPGCGNTGSFNSFCC	>*Bacillus thuringiensis* YBT 1518 (I)<br>ATGATTAATGAAAAAAACCTTTTTGATTTAGATGTACAAGTAACAACAGCTACAGGCGATGTAGATCCACAAATTACAAGTATCTCAGCTTGTACGCCTGGTTGTGGAAACACAGGTTCATTCAATAGCTTCTGTTGCTAA	NC_022873	3953881	3953741
>*Bacillus thuringiensis* YBT 1518 (II)<br>mineknlfdldvqvttatgdvdpqITRISACTPGCGNTGSFNSFCC	>*Bacillus thuringiensis* YBT 1518 (II)<br>ATGATTAATGAAAAAAACCTTTTTGATTTAGATGTACAAGTAACAACAGCTACAGGTGATGTAGATCCACAAATTACAAGAATCTCAGCTTGTACGCCTGGTTGTGGAAACACAGGTTCATTCAATAGCTTCTGTTGCTAA	NC_022873	3954071	3953931
>*Geobacillus kaustophilus* HTA426 (I)<br>maklddfdldivvkkqdnivqpnITSKSLCTPGCITGILMCLTQNSCVSCNSCIRC	>*Geobacillus kaustophilus* HTA426 (I)<br>ATGGCAAAGTTGGATGATTTCGATTTGGACATTGTTGTCAAAAAACAGGACAACATTGTACAACCTAATATTACAAGTAAATCTCTTTGTACCCCGGGTTGCATCACAGGAATCTTGATGTGCTTAACGCAAAATTCTTGTGTATCTTGTAACTCTTGCATTCGATGCTAA	NC_006510	314934	315104
>*Geobacillus kaustophilus* HTA426 (II)<br>maklddfdldivvkkqdnivqpnITSKSLLYPGLHHRHLNVLNAKFLCIL	>*Geobacillus kaustophilus* HTA426 (II)<br>ATGGCGAAGTTGGATGATTTCGATTTGGACATTGTTGTCAAAAAACAGGACAACATTGTACAACCTAATATTACAAGTAAATCTCTTTTGT	NC_006510	320984 [1]	321136 [1]

	ACCCCGGGTTGCATCACAGGCACCTTAATG<br>TGCTTAACGCAAAATTCTTGTGTATCTTGTA<br>A			
**>*Geobacillus* sp. ZGt-1**<br>**maklddfdldivvkkqdnivqpnITSKSLCTPGCITG**<br>**ILMCLTQNSCVSCNSCIRC**	**>*Geobacillus* sp. ZGt-1**<br>**ATGGCAAAGTTGGATGATTTCGATTTGG**<br>**ACATTGTTGTCAAAAAACAGGACAACAT**<br>**TGTACAACCTAATATTACAAGTAAATCTC**<br>**TTTGTACCCCGGGTTGCATCACAGGAATC**<br>**TTGATGTGCTTAACGCAAAATTCTTGTGT**<br>**ATCTTGTAACTCTTGCATTCGATGCTAA**	**LDPD01000000 [2]**	**33624**	**33794**
>*Geobacillus thermodenitrificans* NG80-2<br>makfddfdldivvkkqddvvqpnVTSKSLCTPGCITG<br>VLMCLTQNSCVSCNSCIRC	>*Geobacillus thermodenitrificans* NG80-2<br>ATGGCGAAGTTTGATGATTTTGATTTGGAC<br>ATTGTGGTGAAAAAACAGGACGACGTGGT<br>ACAACCGAACGTGACGAGTAAATCTCTTTG<br>TACGCCAGGTTGTATCACAGGTGTGTTAAT<br>GTGTCTCACGCAAAATTCTTGTGTATCTTGT<br>AACTCTTGCATTCGATGCTAA	NC_009328	298823	298993
>*Geobacillus thermoleovorans* CCB_US3_UF5<br>maklddfdldivvkkqdnivqpnITSKSLLYPGLHHR<br>HLNVLNAKFLCIL	>*Geobacillus thermoleovorans* CCB_US3_UF5<br>ATGGCGAAGTTGGATGATTTCGATTTGGAC<br>ATTGTTGTCAAAAAACAGGACAACATTGTA<br>CAACCTAATATTACAAGTAAATCTCTTTTGT<br>ACCCCGGGTTGCATCACAGGCATCTTAATG<br>TGCTTAACGCAAAATTCTTGTGTATCTTGTA<br>A	NC_016593	362226 [1]	362378 [1]
>*Lactococcus lactis* CV56<br>mstkdfnldlvsvskkdsgasprITSISLCTPGCKTGAL<br>MGCNMKTATCHCSIHVSK	>*Lactococcus lactis* CV56<br>ATGAGTACAAAAGATTTTAACTTGGATTTG<br>GTATCTGTTTCGAAGAAAGATTCAGGTGCA<br>TCACCACGCATTACAAGTATTTCGCTATGT<br>ACACCCGGTTGTAAAACAGGAGCTCTGAT<br>GGGTTGTAACATGAAAACAGCAACTTGTC<br>ATTGTAGTATTCACGTAAGCAAATAA	NC_017486	604687	604860
>*Lactococcus lactis* IO<br>mstkdfnldlvsvskkdsgasprITSISLCTPGCKTGAL<br>MGCNMKTATCNCSIHVSK	>*Lactococcus lactis* IO<br>ATGAGTACAAAAGATTTTAACTTGGATTTG<br>GTATCTGTTTCGAAGAAAGATTCAGGTGCA<br>TCACCACGCATTACAAGTATTTCGCTATGT<br>ACACCCGGTTGTAAAACAGGAGCTCTGAT<br>GGGTTGTAACATGAAAACAGCAACTTGTA<br>ATTGTAGTATTCACGTAAGCAAATAA	NC_020450	599615	599788
>*Paenibacillus polymyxa* CR1<br>mknqfdldlqvaknevapkgvqpaSGII<br>CTPSCATGTLNCQVSLTFCKTC	>*Paenibacillus polymyxa* CR1<br>ATGAAAAATCAATTTGATCTGGACTTGCAA<br>GTAGCTAAAAATGAGGTTGCTCCAAAAGG<br>AGTTCAACCAGCCAGTGGTATTATCTGTAC<br>TCCTTCATGTGCAACAGGTACTTTGAATTGT<br>CAAGTTAGTCTTACCTTCTGCAAAACGTGT<br>TAA	NC_023037	1748588	1748740
>*Paenibacillus polymyxa* E681<br>mknqfdldlqvaknevaskgvqpaSGLICTPSCATGTL<br>NCQVSLSFCKTC	>*Paenibacillus polymyxa* E681<br>ATGAAAAATCAATTTGATCTGGACTTGCAA<br>GTAGCTAAAAATGAGGTTGCTTCAAAAGG<br>GGTTCAACCAGCCAGTGGTCTTATCTGTAC<br>TCCTTCATGTGCAACAGGTACTTTGAATTGT<br>CAAGTTAGTCTTAGCTTCTGCAAAACGTGT<br>TAA	NC_014483	1558420	1558572
**>*Paenibacillus polymyxa* M1 (I)**<br>**mknqfdldlqvtksesaskelqadSGIICTPTCLTSIL**<br>**NCYTSISHCGPC**	**>*Paenibacillus polymyxa* M1 (I)**<br>**ATGAAAAATCAATTTGATCTGGATTTGCA**<br>**AGTGACCAAAAGTGAGAGTGCTTCTAAA**<br>**GAGCTTCAAGCAGACAGCGGCATTATTT**<br>**GTACTCCTACTTGTTTAACATCTATTTTAA**<br>**ACTGCTATACTAGCATTTCACATTGTGGA**<br>**CCTTGTTGA**	**NC_017542**	**1693817**	**1693969**

**>*Paenibacillus polymyxa* M1 (II)**<br>**mknqfdldlqvaknevapkevqpaSGLICTPSCATG<br>TLNCQVSLSFCKTC**	**>*Paenibacillus polymyxa* M1 (II)**<br>**ATGAAAAATCAATTTGATCTGGACTTGCA<br>AGTAGCCAAAAATGAGGTTGCTCCTAAA<br>GAGGTTCAACCAGCTAGCGGTCTTATCT<br>GTACTCCTTCCTGTGCAACAGGTACTTTG<br>AATTGCCAAGTTAGCCTTTCCTTCTGCAA<br>AACGTGTTAA**	**NC_017542**	**1695699**	**1695851**
**>*Paenibacillus polymyxa* SC2 (I)**<br>**mknqfdldlqvtksesaskelqadSGIICTPTCLTSIL<br>NCYTSISHCGPC**	**>*Paenibacillus polymyxa* SC2 (I)**<br>**ATGAAAAATCAATTTGATCTGGATTTGCA<br>AGTGACCAAAAGTGAGAGTGCTTCTAAA<br>GAGCTTCAAGCAGACAGCGGCATTATTT<br>GTACTCCTACTTGTTTAACATCTATTTTAA<br>ACTGCTATACTAGCATTTCACATTGTGGA<br>CCTTGTTGA**	**NC_014622**	**1695159**	**1695311**
**>*Paenibacillus polymyxa* SC2 (II)**<br>**mknqfdldlqvaknevapkevqpaSGLICTPSCATG<br>TLNCQVSLSFCKTC**	**>*Paenibacillus polymyxa* SC2 (II)**<br>**ATGAAAAATCAATTTGATCTGGACTTGCA<br>AGTAGCCAAAAATGAGGTTGCTCCTAAA<br>GAGGTTCAACCAGCTAGCGGTCTTATCT<br>GTACTCCTTCCTGTGCAACAGGTACTTTG<br>AATTGCCAAGTTAGCCTTTCCTTCTGCAA<br>AACGTGTTAA**	**NC_014622**	**1697041**	**1697193**
>*Staphylococcus aureus* 11819-97 (I)<br>mekvldldvqvkannnsndsagdeRITSHSLCTPGCA<br>KTGSFNSFCC	>*Staphylococcus aureus* 11819-97 (I)<br>ATGGAAAAAGTTCTTGATTTAGACGTGCAA<br>GTTAAAGCAAACAATAACTCAAATGATTC<br>AGCAGGTGACGAACGTATTACAAGTCATA<br>GTTTATGTACTCCTGGTTGTGCTAAGACTGG<br>TAGTTTTAATAGCTTCTGCTGTTAA	NC_017351	1942472	1942329
>*Staphylococcus aureus* 11819-97 (II)<br>mekvldldvqvkgnnntndsagdeRITSHLFCSFGCEK<br>TGSFNSFCC	>*Staphylococcus aureus* 11819-97 (II)<br>TTGGAAAAAGTTCTTGATTTAGACGTGCAA<br>GTTAAAGGAAATAACAACACTAATGATTC<br>AGCGGGTGACGAAAGAATAACTAGCCATC<br>TTTTTTGTAGCTTTGGTTGTGAAAAGACGGG<br>TAGTTTTAACAGCTTCTGTTGTTAA	NC_017351	1943352	1943209
>*Staphylococcus aureus* Bmb9393 (I)<br>mekvldldvqvkannnsndsagdeRITSHSLCTPGCA<br>KTGSFNSFCC	>*Staphylococcus aureus* Bmb9393 (I)<br>ATGGAAAAAGTTCTTGATTTAGACGTGCAA<br>GTTAAAGCAAACAATAACTCAAATGATTC<br>AGCAGGTGACGAACGTATTACAAGTCATA<br>GTTTATGTACTCCTGGTTGTGCTAAGACTGG<br>TAGTTTTAATAGCTTCTGCTGTTAA	NC_021670	2038104	2037961
>*Staphylococcus aureus* Bmb9393 (II)<br>mekvldldvqvkgnnntndsagdeRITSHLFCSFGCEK<br>TGSFNSFCC	> *Staphylococcus aureus* Bmb9393 (II)<br>TTGGAAAAAGTTCTTGATTTAGACGTGCAA<br>GTTAAAGGAAATAACAACACTAATGATTC<br>AGCGGGTGACGAAAGAATAACTAGCCATC<br>TTTTTTGTAGCTTTGGTTGTGAAAAGACGGG<br>TAGTTTTAACAGCTTCTGTTGTTAA	NC_021670	2038984	2038841
>*Staphylococcus aureus* COL (I)<br>mekvldldvqvkannnsndsagdeRITSHSLCTPGCA<br>KTGSFNSFCC	>*Staphylococcus aureus* COL (I)<br>ATGGAAAAAGTTCTTGATTTAGACGTGCAA<br>GTTAAAGCAAACAATAACTCAAATGATTC<br>AGCAGGTGACGAACGTATTACAAGTCATA<br>GTTTATGTACTCCTGGTTGTGCTAAGACTGG<br>TAGTTTTAATAGCTTCTGCTGTTAA	NC_002951	1932028	1931885
>*Staphylococcus aureus* COL (II)<br>mekvldldvqvkgnnntndsagdeRITSHLFCSFGCEK<br>TVLHQMWWVYNLKNYF	> *Staphylococcus aureus* COL (II)<br>TTGGAAAAAGTTCTTGATTTAGACGTGCAA<br>GTTAAAGGAAATAACAACACTAATGATTC<br>AGCGGGTGACGAAAGAATAACTAGCCATC<br>TTTTTTGTAGCTTTGGTTGTGAAAAGACGGT<br>TCTCCACCAAATGTGGTGGGTATATAATTT<br>AAAGAACTATTTTTAA	NC_002951	1934429	1934265

>*Staphylococcus aureus* ED133 (I)<br>menvldldvqvkakndtsdsaGDERITSFIGCTPGCG<br>KTGSFNSFCC	>*Staphylococcus aureus* ED133 (I)<br>ATGGAAAATGTTCTTGATTTAGACGTACAA<br>GTAAAAGCGAAAAACGACACTTCTGATTC<br>AGCGGGTGACGAACGTATTACTAGTTTCAT<br>TGGTTGTACTCCTGGTTGTGGTAAGACTGGT<br>AGTTTTAATAGCTTCTGCTGTTAA	NC_017337	1905359	1905216
>*Staphylococcus aureus* ED133 (II)<br>mekvldldvqvkgnnntndsaaderITSHFLCSFGCGK<br>TGSFNSFCC	>*Staphylococcus aureus* ED133 (II)<br>ATGGAAAAAGTTCTTGATTTAGACGTGCAA<br>GTTAAAGGAAATAACAACACTAATGATTC<br>AGCGGCTGACGAAAGAATAACTAGCCATT<br>TTTTATGTAGCTTTGGTTGTGGAAAGACGG<br>GTAGTTTTAACAGCTTCTGTTGTTAA	NC_017337	1907901	1907758
>*Staphylococcus aureus* LGA251 (I)<br>menvldldvqvkakndtsdsaGDERITSFIGCTPGCG<br>KTGSFNSFCC	>*Staphylococcus aureus* LGA251 (I)<br>ATGGAAAATGTTCTTGATTTAGACGTACAA<br>GTAAAAGCGAAAAACGACACTTCTGATTC<br>AGCGGGTGACGAACGTATTACTAGTTTCAT<br>TGGTTGTACTCCTGGTTGTGGTAAGACTGGT<br>AGTTTTAATAGCTTCTGCTGTTAA	NC_017349	1893217	1893074
>*Staphylococcus aureus* LGA251 (II)<br>mekvldldvqvkgnnntndsaaderITSHFLCSFGCGK<br>TGSFNSFCC	>*Staphylococcus aureus* LGA251 (II)<br>ATGGAAAAAGTTCTTGATTTAGACGTGCAA<br>GTTAAAGGAAATAACAACACTAATGATTC<br>AGCGGCTGACGAAAGAATAACTAGCCATT<br>TTTTATGTAGCTTTGGTTGTGGAAAGACGG<br>GTAGTTTTAACAGCTTCTGTTGTTAA	NC_017349	1894096	1893953
>*Staphylococcus aureus* M1 (I)<br>mekvldldvqvkannnsndsagdeRITSHSLCTPGCA<br>KTGSFNSFC	>*Staphylococcus aureus* M1 (I)<br>ATGGAAAAAGTTCTTGATTTAGACGTGCAA<br>GTTAAAGCAAACAATAACTCAAATGATTC<br>AGCAGGTGACGAACGTATTACAAGTCATA<br>GTTTATGTACTCCTGGTTGTGCTAAGACTGG<br>TAGTTTTAATAGCTTCTGCTGTTAA	NC_021059	1944795	1944652
>*Staphylococcus aureus* M1 (II)<br>mekvldldvqvkgnnntndsagdeRITSHLFCSFGCEK<br>TGSFNSFCC	>*Staphylococcus aureus* M1 (II)<br>TTGGAAAAAGTTCTTGATTTAGACGTGCAA<br>GTTAAAGGAAATAACAACACTAATGATTC<br>AGCGGGTGACGAAAGAATAACTAGCCATC<br>TTTTTTGTAGCTTTGGTTGTGAAAAGACGGG<br>TAGTTTTAACAGCTTCTGTTGTTAA	NC_021059	1945675	1945532
>*Staphylococcus_aureus* MSSA476 (I)<br>mekvldldvqvkannnsndsagdeRITSHSLCTPGCA<br>KTGSFNSFCC	> *Staphylococcus_aureus* MSSA476 (I)<br>ATGGAAAAAGTTCTTGATTTAGACGTGCAA<br>GTTAAAGCAAACAATAACTCAAATGATTC<br>AGCAGGTGACGAACGTATTACAAGTCATA<br>GTTTATGTACTCCTGGTTGTGCTAAGACTGG<br>TAGTTTTAATAGCTTCTGCTGTTAA	NC_002953	1896691	1896548
>*Staphylococcus_aureus* MSSA476 (II)<br>mekvldldvqvkgnnntndsagdeRITSHLFCSFGCEK<br>TGSFNSFCC	>*Staphylococcus_aureus* MSSA476 (II)<br>TTGGAAAAAGTTCTTGATTTAGACGTGCAA<br>GTTAAAGGAAATAACAACACTAATGATTC<br>AGCGGGTGACGAAAGAATAACTAGCCATC<br>TTTTTTGTAGCTTTGGTTGTGAAAAGACGGG<br>TAGTTTTAACAGCTTCTGTTGTTAA	NC_002953	1897571	1897428
>*Staphylococcus aureus* MW2 (I)<br>mekvldldvqvkannnsndsagdeRITSHSLCTPGCA<br>KTGSFNSFCC	>*Staphylococcus aureus* MW2 (I)<br>ATGGAAAAAGTTCTTGATTTAGACGTGCAA<br>GTTAAAGCAAACAATAACTCAAATGATTC<br>AGCAGGTGACGAACGTATTACAAGTCATA<br>GTTTATGTACTCCTGGTTGTGCTAAGACTGG<br>TAGTTTTAATAGCTTCTGCTGTTAA	NC_003923	1917357	1917214
>*Staphylococcus aureus* MW2 (II)<br>mekvldldvqvkgnnntndsagdeRITSHLFCSFGCEK<br>TGSFNSFCC	>*Staphylococcus aureus* MW2 (II)<br>TTGGAAAAAGTTCTTGATTTAGACGTGCAA<br>GTTAAAGGAAATAACAACACTAATGATTC<br>AGCGGGTGACGAAAGAATAACTAGCCATC<br>TTTTTTGTAGCTTTGGTTGTGAAAAGACGGG	NC_003923	1918237	1918094

	TAGTTTTAACAGCTTCTGTTGTTAA			
>*Staphylococcus aureus* NCTC 8325 (I)<br>mekvldldvqvkannnsndsagdeRITSHSLCTPGCAKTGSFNSFCC	>*Staphylococcus aureus* NCTC 8325 (I)<br>ATGGAAAAAGTTCTTGATTTAGACGTGCAAGTTAAAGCAAACAATAACTCAAATGATTCAGCAGGTGACGAACGTATTACAAGTCATAGTTTATGTACTCCTGGTTGTGCTAAGACTGGTAGTTTTAATAGCTTCTGCTGTTAA	NC_007795	1857888	1857745
**>*Staphylococcus aureus* NCTC 8325 (II)<br>lekvldldvqvkgnnntndsagdeRITSHLFCSFGCEKTGSFNSFCC**	**>*Staphylococcus aureus* NCTC 8325 (II)<br>TTGGAAAAAGTTCTTGATTTAGACGTGCAAGTTAAAGGAAATAACAACACTAATGATTCAGCGGGTGACGAAAGAATAACTAGCCATCTTTTTTGTAGCTTTGGTTGTGAAAAGACGGGTAGTTTTAACAGCTTCTGTTGTTAA**	**NC_007795**	**1858768**	**1858625**
>*Staphylococcus aureus* Newman (I)<br>mekvldldvqvkannnsndsagdeRITSHSLCTPGCAKTGSFNSFCC	>*Staphylococcus aureus* Newman (I)<br>ATGGAAAAAGTTCTTGATTTAGACGTGCAAGTTAAAGCAAACAATAACTCAAATGATTCAGCAGGTGACGAACGTATTACAAGTCATAGTTTATGTACTCCTGGTTGTGCTAAGACTGGTAGTTTTAATAGCTTCTGCTGTTAA	NC_009641	1914736	1914593
>*Staphylococcus aureus* Newman (II)<br>mekvldldvqvkgnnntndsagdeRITSHLFCSFGCEKTGSFNSFCC	>*Staphylococcus aureus* Newman (II)<br>TTGGAAAAAGTTCTTGATTTAGACGTGCAAGTTAAAGGAAATAACAACACTAATGATTCAGCGGGTGACGAAAGAATAACTAGCCATCTTTTTTGTAGCTTTGGTTGTGAAAAGACGGGTAGTTTTAACAGCTTCTGTTGTTAA	NC_009641	1915616	1915473
>*Staphylococcus aureus* RF122 (I)<br>menvldldvqvkakndtsdsaGDERITSFIGCTPGCGKTGSFNSFCC	>*Staphylococcus aureus* RF122 (I)<br>ATGGAAAATGTTCTTGATTTAGACGTACAAGTAAAAGCGAAAAACGACACTTCTGATTCAGCGGGTGACGAACGTATTACTAGTTTCATTGGTTGTACTCCTGGTTGTGGTAAGACTGGTAGTTTTAATAGCTTCTGCTGTTAA	NC_007622	1829509	1829366
>*Staphylococcus aureus* RF122 (II)<br>mekvldldvqvkgnnntndsagdeRITSHFLCSFGCGKTGSFNSFCC	>*Staphylococcus aureus* RF122 (II)<br>ATGGAAAAAGTTCTTGATTTAGACGTGCAAGTTAAAGGAAATAACAACACTAATGATTCAGCGGGTGACGAAAGAATAACTAGCCATTTTTTATGTAGCTTTGGTTGTGGAAAGACGGGTAGTTTTAACAGCTTCTGTTGTTAA	NC_007622	1830386	1830243
>*Staphylococcus aureus* T0131 (I)<br>mekvldldvqvkannnsndsagdeRITSHSLCTPGCAKTGSFNSFCC	>*Staphylococcus aureus* T0131 (I)<br>ATGGAAAAAGTTCTTGATTTAGACGTGCAAGTTAAAGCAAACAATAACTCAAATGATTCAGCAGGTGACGAACGTATTACAAGTCATAGTTTATGTACTCCTGGTTGTGCTAAGACTGGTAGTTTTAATAGCTTCTGCTGTTAA	NC_017347	1932461	1932318
>*Staphylococcus aureus* T0131 (II)<br>mekvldldvqvkgnnntndsagdeRITSHLFCSFGCEKTGSFNSFCC	>*Staphylococcus aureus* T0131 (II)<br>TTGGAAAAAGTTCTTGATTTAGACGTGCAAGTTAAAGGAAATAACAACACTAATGATTCAGCGGGTGACGAAAGAATAACTAGCCATCTTTTTTGTAGCTTTGGTTGTGAAAAGACGGGTAGTTTTAACAGCTTCTGTTGTTAA	NC_017347	1933341	1933198
>*Staphylococcus aureus* TW20 (I)<br>mekvldldvqvkannnsndsagdeRITSHSLCTPGCAKTGSFNSFCC	>*Staphylococcus aureus* TW20 (I)<br>ATGGAAAAAGTTCTTGATTTAGACGTGCAAGTTAAAGCAAACAATAACTCAAATGATTCAGCAGGTGACGAACGTATTACAAGTCATAGTTTATGTACTCCTGGTTGTGCTAAGACTGGTAGTTTTAATAGCTTCTGCTGTTAA	NC_017331	1978007	1977864
>*Staphylococcus aureus* TW20 (II)<br>mekvldldvqvkgnnntndsagdeRITSHLFCSFGCEKTGSFNSFCC	>*Staphylococcus aureus* TW20 (II)<br>TTGGAAAAAGTTCTTGATTTAGACGTGCAAGTTAAAGGAAATAACAACACTAATGATTC	NC_017331	1978887	1978744

	AGCGGGTGACGAAAGAATAACTAGCCATC<br>TTTTTTGTAGCTTTGGTTGTGAAAAGACGGG<br>TAGTTTTAACAGCTTCTGTTGTTAA			
>*Staphylococcus aureus* USA300 FPR3757 (I)<br>mekvldldvqvkannnsndsagdeRITSHSLCTPGCA<br>KTGSFNSFCC	>*Staphylococcus aureus* USA300 FPR3757(I)<br>ATGGAAAAAGTTCTTGATTTAGACGTGCAA<br>GTTAAAGCAAACAATAACTCAAATGATTC<br>AGCAGGTGACGAACGTATTACAAGTCATA<br>GTTTATGTACTCCTGGTTGTGCTAAGACTGG<br>TAGTTTTAATAGCTTCTGCTGTTAA	NC_007793	1954197	1954054
>*Staphylococcus aureus* USA300 FPR3757 (II)<br>mekvldldvqvkgnnntndsagdeRITSHLFCSFGCEK<br>TGSFNSFCC	>*Staphylococcus aureus* USA300 FPR3757 (II)<br>TTGGAAAAAGTTCTTGATTTAGACGTGCAA<br>GTTAAAGGAAATAACAACACTAATGATTC<br>AGCGGGTGACGAAAGAATAACTAGCCATC<br>TTTTTTGTAGCTTTGGTTGTGAAAAGACGGG<br>TAGTTTTAACAGCTTCTGTTGTTAA	NC_007793	1955077	1954934
>*Staphylococcus aureus* USA300_TCH1516 (I)<br>mekvldldvqvkannnsndsagdeRITSHSLCTPGCA<br>KTGSFNSFCC	>*Staphylococcus aureus* USA300_TCH1516 (I)<br>ATGGAAAAAGTTCTTGATTTAGACGTGCAA<br>GTTAAAGCAAACAATAACTCAAATGATTC<br>AGCAGGTGACGAACGTATTACAAGTCATA<br>GTTTATGTACTCCTGGTTGTGCTAAGACTGG<br>TAGTTTTAATAGCTTCTGCTGTTAA	NC_010079	1954946	1954803
>*Staphylococcus aureus* USA300_TCH1516 (II)<br>mekvldldvqvkgnnntndsagdeRITSHLFCSFGCEK<br>TGSFNSFCC	>*Staphylococcus aureus* USA300_TCH1516 (II)<br>TTGGAAAAAGTTCTTGATTTAGACGTGCAA<br>GTTAAAGGAAATAACAACACTAATGATTC<br>AGCGGGTGACGAAAGAATAACTAGCCATC<br>TTTTTTGTAGCTTTGGTTGTGAAAAGACGGG<br>TAGTTTTAACAGCTTCTGTTGTTAA	NC_010079	1955826	1955683
>*Staphylococcus aureus* VC40 (I)<br>mekvldldvqvkannnsndsagdeRITSHSLCTPGCA<br>KTGSFNSFCC	>*Staphylococcus aureus* VC40 (I)<br>ATGGAAAAAGTTCTTGATTTAGACGTGCAA<br>GTTAAAGCAAACAATAACTCAAATGATTC<br>AGCAGGTGACGAACGTATTACAAGTCATA<br>GTTTATGTACTCCTGGTTGTGCTAAGACTGG<br>TAGTTTTAATAGCTTCTGCTGTTAA	NC_016912	1815518	1815375
>*Staphylococcus aureus* VC40 (II)<br>mekvldldvqvkgnnntndsagdeRITSHLFCSFGCEK<br>TGSFNSFCC	> *Staphylococcus aureus* VC40 (II)<br>TTGGAAAAAGTTCTTGATTTAGACGTGCAA<br>GTTAAAGGAAATAACAACACTAATGATTC<br>AGCGGGTGACGAAAGAATAACTAGCCATC<br>TTTTTTGTAGCTTTGGTTGTGAAAAGACGGG<br>TAGTTTTAACAGCTTCTGTTGTTAA	NC_016912	1816398	1816255
>*Staphylococcus aureus* Z172 (I)<br>mekvldldvqvkannnsndsagdeRITSHSLCTPGCA<br>KTGSFNSFCC	>*Staphylococcus aureus* Z172 (I)<br>ATGGAAAAAGTTCTTGATTTAGACGTGCAA<br>GTTAAAGCAAACAATAACTCAAATGATTC<br>AGCAGGTGACGAACGTATTACAAGTCATA<br>GTTTATGTACTCCTGGTTGTGCTAAGACTGG<br>TAGTTTTAATAGCTTCTGCTGTTAA	NC_022604	1976108	1975965
>*Staphylococcus aureus* Z172 (II)<br>mekvldldvqvkgnnntndsagdeRITSHLFCSFGCEK<br>TGSFNSFCC	>*Staphylococcus aureus* Z172 (II)<br>TTGGAAAAAGTTCTTGATTTAGACGTGCAA<br>GTTAAAGGAAATAACAACACTAATGATTC<br>AGCGGGTGACGAAAGAATAACTAGCCATC<br>TTTTTTGTAGCTTTGGTTGTGAAAAGACGGG<br>TAGTTTTAACAGCTTCTGTTGTTAA	NC_022604	1978319	1978176
**>*Streptococcus intermedius* B196<br>msnhddfqldtnkvnfdndqevrGPLSWLGGTVLT<br>SIVTGCTGDCLTRHCADNVTKNTGCTVTH<br>PRCKR**	**>*Streptococcus intermedius* B196<br>ATGTCAAATCATGATGATTTTCAATTAGA<br>TACAAACAAAGTTAATTTTGATAATGATC<br>AAGAAGTAAGAGGTCCGCTTTCTTGGCT<br>AGGCGGAACAGTATTAACTTCAATAGTT<br>ACAGGCTGCACAGGAGATTGTTTAACAA<br>GGCACTGTGCCGATAATGTTACAAAAAA<br>TACAGGCTGTACAGTTACGCATCCTAGAT**	**NC_022246**	**590584**	**590793**

	**GTAAAAGGTAG**			
>*Streptococcus intermedius* C270<br>mstkdfnldlvsvsktdagasprVTSVFMCTPGCVTGALMGCNLQTATCNCHVHISK	>*Streptococcus intermedius* C270<br>ATGAGTACGAAAGATTTTAACTTAGATTTGGTTTCTGTTTCAAAAACAGATGCTGGTGCTTCACCTCGTGTTACTAGTGTGTTTATGTGTACTCCAGGTTGTGTAACTGGTGCTTTGATGGGTTGTAACTTGCAAACAGCTACATGTAACTGTCATGTTCACATTAGTAAATAA	NC_022237	1010608	1010435
>*Streptococcus pasteurianus* ATCC 43144<br>mnnedfnldlvtiskennsgasprVTSKSLCTPGCKTGILMTCAIKTATCGCHFG	>*Streptococcus pasteurianus* ATCC 43144<br>ATGAACAATGAAGATTTTAATTTGGATCTTGTCACAATCTCAAAGGAAAACAACTCAGGAGCTTCACCTCGGGTAACTAGTAAATCATTATGTACTCCTGGATGTAAGACGGGTATTTTGATGACCTGTGCAATCAAAACTGCAACTTGTGGTTGCCATTTTGGATAA	NC_015600	1148270	1148103
>*Streptococcus pyogenes* MGAS6180<br>mnntikdfdldlktnkkdtatpyVGSRYLCTPGSCWKLVCFTTTVK	>*Streptococcus pyogenes* MGAS6180<br>ATGAATAACACAATTAAAGACTTTGATCTCGATTTGAAAACAAATAAAAAAGACACTGCTACACCTTATGTTGGTAGCCGTTACCTATGTACCCCTGGTTCTTGTTGGAAATTAGTTTGCTTTACAACAACTGTTAAATAA	NC_007296	804231	804371
>*Streptococcus pyogenes* MGAS9429<br>mnntikdfdldlktnkkdtatpyVGSRYLCTPGSCWKLVCFTTTVK	>*Streptococcus pyogenes* MGAS9429<br>ATGAATAACACAATTAAAGACTTTGATCTCGATTTGAAAACAAATAAAAAAGACACTGCTACACCTTATGTTGGTAGCCGTTACCTATGTACCCCTGGTTCTTGTTGGAAATTAGTTTGCTTTACAACAACTGTTAAATAA	NC_008021	885676	885816
>*Streptococcus pyogenes* MGAS10270<br>mnntikdfdldlktnkkdtatpyVGSRYLCTPGSCWKLVCFTTTVK	>*Streptococcus pyogenes* MGAS10270<br>ATGAATAACACAATTAAAGACTTTGATCTCGATTTGAAAACAAATAAAAAAGACACTGCTACACCTTATGTTGGTAGCCGTTACCTATGTACCCCTGGTTCTTGTTGGAAATTAGTTTGCTTTACAACAACTGTTAAATAA	NC_008022	876632	876772
>*Streptococcus pyogenes* MGAS10750<br>mtntikdfdldlktnkkdtatpyVGSRYLCTPGSCWKLVCFTTTVK	>*Streptococcus pyogenes* MGAS10750<br>ATGACTAACACAATTAAAGACTTTGATCTCGATTTGAAAACAAATAAAAAAGACACTGCTACACCTTATGTTGGTAGCCGTTACCTATGTACCCCTGGTTCTTGTTGGAAATTAGTTTGCTTTACAACAACTGTTAAATAA	NC_008024	907659	907799
>*Streptococcus suis* JS14<br>mnnedfnldlvtiskennsgasprVTSKSLCTPGCKTGILMTCAIKTATCGCHFG	>*Streptococcus suis* JS14<br>ATGAACAATGAAGATTTTAATTTGGATCTTGTCACAATCTCAAAGGAAAACAACTCAGGAGCTTCACCTCGGGTAACTAGTAAATCATTATGTACTCCTGGATGTAAGACGGGTATTTTGATGACCTGTGCAATCAAAACTGCAACTTGTGGTTGCCATTTTGGATAA	NC_017618	646342	646509
>*Streptococcus suis* SC070731<br>mnnedfnldlvtiskennsgasprVTSKSLCTPGCKTGILMTCAIKTATCGCHFG	>*Streptococcus suis* SC070731<br>ATGAACAATGAAGATTTTAATTTGGATCTTGTCACAATCTCAAAGGAAAACAACTCAGGAGCTTCACCTCGGGTAACTAGTAAATCATTATGTACTCCTGGATGTAAGACGGGTATTTTGATGACCTGTGCAATCAAAACTGCAACTTGTGGTTGCCATTTTGGATAA	NC_020526	650359	650526

[1] The presented gene positions are based on those predicted by antiSMASH 3.0 and confirmed by BLAST analysis.

[2] The presented genome sequence accession number for Geobacillus sp. ZGt-1 belongs to the original genome record; the whole genome shotgun (WGS) project.

Table S.2. The antiSMASH-predicted lanthipeptides whose coding genes have either been unannotated, inaccurately annotated, or annotated as coding for "hypothetical" proteins or generally as "antibiotics".

Bacterial species, strain (lanthipeptide reference number) [1]	Refseq genome accession number	Annotation of the predicted lanthipeptide		Identity to experimentally verified lanthipeptide [2]	Reference [3]
		Original genome record	RefSeq genome record		
Bacillus clausii KSM-K16	NC_006582	Unannotated	"Gallidermin/nisin family lantibiotic"	56% clausin	[30], and this study
Bacillus megaterium QM B1551 (I)	NC_014023	"Antibiotic protein, putative"	"Gallidermin/nisin family lantibiotic"	56% gallidermin	[31], and this study
Bacillus megaterium QM B1551 (II)	NC_014023	"Antibiotic protein, putative"	"Gallidermin/nisin family lantibiotic"	56% gallidermin	[31], and this study
Bacillus subtilis BSn5	NC_014976	"Hypothetical"	"Hypothetical"	100% subtilomycin	[32], and this study
Bacillus subtilis spizizenii W23	NC_014479	"Hypothetical"	"Gallidermin/nisin family lantibiotic"	100% subtilin	This study
Bacillus thuringiensis serovar finitimus YBT-020	NC_017200	"Hypothetical"	Unannotated	No hits	This study
Bacillus thuringiensis serovar IS5056 (I)	NC_020394	"Hypothetical"	"Hypothetical"	100% thuricin 4A-4	[31], and this study
Bacillus thuringiensis serovar IS5056 (II)	NC_020394	"Hypothetical"	"Hypothetical"	100% thuricin 4A-4	[31], and this study
Bacillus thuringiensis serovar IS5056 (III)	NC_020394	"Hypothetical"	"Hypothetical"	86% thuricin 4A-4	[31], and this study
Bacillus thuringiensis serovar IS5056 (IV)	NC_020394	"Hypothetical"	"Hypothetical"	84% thuricin 4A-4	[31], and this study
Bacillus thuringiensis serovar IS5056 (V)	NC_020394	"Hypothetical"	"Hypothetical"	82% thuricin 4A-4	[31], and this study
Bacillus thuringiensis YBT 1518 (I)	NC_022873	"Antibiotic protein, putative"	"Gallidermin/nisin family lantibiotic"	53% gallidermin	This study
Bacillus thuringiensis YBT 1518 (II)	NC_022873	"Antibiotic protein, putative"	"Gallidermin/nisin family lantibiotic"	51% gallidermin	This study
Geobacillus thermoleovorans CCB_US3_UF5	NC_016593	Unannotated	Partly inaccurately annotated gene	79% geobacillin I	This study
Paenibacillus polymyxa CR1	NC_023037	"Subtilin lantibiotic" [4]	"Hypothetical"	94% paenilan	[36, 37], and this study
Paenibacillus polymyxa E681	NC_014483	"Subtilin lantibiotic" [4]	"Gallidermin/nisin family lantibiotic"	100% paenilan	[36], and this study
Paenibacillus polymyxa M1 (I)	NC_017542	Unannotated	"Hypothetical"	64% paenilan	This study
Paenibacillus polymyxa M1 (II)	NC_017542	Unannotated	"Hypothetical"	96% paenilan	This study
Paenibacillus polymyxa SC2 (I)	NC_014622	Partly inaccurately annotated gene	"Hypothetical"	64% paenilan	[30], and this study
Paenibacillus polymyxa SC2 (II)	NC_014622	"Subtilin lantibiotic" [4]	"Hypothetical"	96% paenilan	This study
Staphylococcus aureus 11819-97 (II)	NC_017351	Unannotated	"Gallidermin/nisin family lantibiotic"	83% BsaA2	This study
Staphylococcus aureus COL (II)	NC_002951	Unannotated	"Gallidermin/nisin family lantibiotic"	79% BsaA2	This study
Staphylococcus aureus ED133 (I)	NC_017337	"Hypothetical"	"Gallidermin/nisin family lantibiotic"	100% BacCH91	This study
Staphylococcus aureus ED133 (II)	NC_017337	"Hypothetical"	"Gallidermin/nisin family lantibiotic"	85% BsaA2	[30], and this study
Staphylococcus aureus M1 (II)	NC_021059	Unannotated	"Gallidermin/nisin family lantibiotic"	83% BsaA2	This study
Staphylococcus aureus MSSA476 (I)	NC_002953	"Putative	"Gallidermin/nisin	100% BsaA2	[8], and this

		isochorismatase"	family lantibiotic"		study
Staphylococcus aureus NCTC 8325 (II)	NC_007795	Unannotated	Unannotated	83% BsaA2	This study
Staphylococcus aureus RF122 (I)	NC_007622	"Hypothetical"	"Gallidermin/nisin family lantibiotic"	100% BacCH91	[15], and this study
Staphylococcus aureus RF122 (II)	NC_007622	"Hypothetical"	"Gallidermin/nisin family lantibiotic"	87% identical to BsaA2	[8], and this study
Staphylococcus aureus T0131 (II)	NC_017347	Unannotated	"Gallidermin/nisin family lantibiotic"	83% BsaA2	This study
Staphylococcus aureus USA300 FPR3757 (II)	NC_007793	Unannotated	"Gallidermin/nisin family lantibiotic"	83% BsaA2	This study
Staphylococcus aureus USA300_TCH1516 (II)	NC_010079	Unannotated	"Gallidermin/nisin family lantibiotic"	83% BsaA2	This study
Staphylococcus aureus Z172 (II)	NC_022604	Unannotated	"Gallidermin/nisin family lantibiotic"	83% BsaA2	This study
Streptococcus intermedius B196	NC_022246	Unannotated	"Hypothetical"	No hits	This study
Streptococcus intermedius C270	NC_022237	"Hypothetical"	"Gallidermin/nisin family lantibiotic"	81% nisin F	This study
Streptococcus pyogenes MGAS9429	NC_008021	Unannotated	"Gallidermin/nisin family lantibiotic"	100% streptin	This study
Streptococcus suis JS14	NC_017618	Unannotated	"Gallidermin/nisin family lantibiotic"	100% suicin 90-1330	This study
Streptococcus suis SC070731	NC_020526	Unannotated	"Gallidermin/nisin family lantibiotic"	100% suicin 90-1330	This study

[1] The lanthipeptide aa sequence is presented in Table S1.

[2] Identity % to experimentally proved lanthipeptides is based on BAGEL BLAST, literature, or both.

[3] The cited references represent studies where the aa sequence of the respective lanthipeptide (or its core peptide) was determined either based on an *in silico* analysis or experimental investigation.

[4] Incorrectly annotated as subtilin. The aa sequences of the lanthipeptides do not match with that of the experimentally confirmed "lantibiotic subtilin" (AAB91589; WP_003220055) produced by *B. subtilis*.

Table S3. BLASTp results of the proteins coded by the Z-geobacillin biosynthesis gene cluster of *Geobacillus* sp. strain ZGt-1. blastp was run against the non-redundant protein sequences (nr) database.

Protein	Gene id [1]	Protein description	Accession number	Identity	Query Coverage	E-value
ZgeoA	6_34	MULTISPECIES: gallidermin/nisin family lantibiotic [Bacillaceae]	WP_011229795	100%	100%	2e-31
ZgeoB	6_44	MULTISPECIES: lantibiotic biosynthesis protein SpaB [*Geobacillus*]	WP_014194788	100%	100%	0.0
ZgeoT	6_45	MULTISPECIES: ABC transporter ATP-binding protein [*Geobacillus*]	WP_025038888	100%	35% [2]	0.0
ZgeoC	6_46	MULTISPECIES: lanthionine synthetase C family protein [*Geobacillus*]	WP_020279663	100%	100%	0.0
ZgeoR	6_47	Two component transcriptional regulator, winged helix [*Geobacillus thermoleovorans* CCB_US3_UF5]	AEV17716.1 [3]	100%	100%	3e-167
ZgeoK	6_48	MULTISPECIES: sensor histidine kinase [Geobacillus]	WP_011229815	100%	100%	0.0
ZgeoI	6_49	MULTISPECIES: NisI/SpaI family lantibiotic immunity lipoprotein [*Geobacillus*]	WP_025039376	100%	100%	8e-102
ZgeoG	6_50	MULTISPECIES: lantibiotic immunity ABC transporter MutG family permease subunit [Bacillaceae]	WP_004888811 [4]	100%	100%	1e-170
ZgeoE	6_52	lantibiotic ABC transporter permease [*Geobacillus thermoleovorans* B23]	GAJ60336 [5]	100%	100%	2e-175
ZgeoF	6_53	MULTISPECIES: lantibiotic ABC transporter ATP-binding protein [*Geobacillus*]	WP_008881431	100%	100%	2e-162

[1] Gene identity is presented as contig, and running number of the predicted genes within the contig. The contig numbers are the same as found in the NCBI genome record (LDPD01000000).

[2] The low matching identity is explained by the 3288 undetermined nucleotides in the gene sequence.

[3] The protein hit has 5 more amino acids at the N-terminal as compared to the antiSMASH 3.0-predicted lanthipeptide.

[4] The protein hit has 20 more amino acids at the N-terminal as compared to the antiSMASH 3.0-predicted lanthipeptide.

[5] The protein hit has 3 more amino acids at the N-terminal as compared to the antiSMASH 3.0-predicted lanthipeptide.

Table S4. Annotation of the proteins coded by the lanthipeptide biosynthesis gene cluster of *G.kaustophilus* HTA426. As described by NCBI, protein accession numbers with the WP_ prefix represent "non-redundant RefSeq protein records" that are "found in RefSeq genomes from multiple species". (~) denotes that the presented protein is similar to the corresponding lanthipeptide cluster protein. Locus-tags containing underscores represent annotations of the RefSeq genome records, while those without underscores were derived from the annotations of the original genome records.

Protein coded by a member of the lanthipeptide gene cluster	Accession number	Locus-tag
LanAI	BAD74571<br>WP_011229795	GK0286<br>GK_RS18085
LanAII	BAD74579<br>~WP_008881441	GK0294<br>GK_RS01950
LanB	BAD74585<br>~WP_014194788	GK0300<br>GK_RS18110
LanBII	~WP_014194788	Not annotated on the original genome<br>In Refseq: GK_RS01970 [1]
LanC	BAD74589<br>WP_041467776	GK0304<br>GK_RS01985
LanT	BAD74588<br>WP_011229812	GK0303<br>GK_RS01980
LanK	BAD74592<br>WP_011229815	GK0307<br>GK_RS01995
LanR	BAD74591<br>WP_004888808	GK0306<br>GK_RS01990
LanI	BAD74594<br>~WP_008881434	GK0309<br>GK_RS02005
LanF	BAD74597<br>WP_008881431	GK0312<br>GK_RS02020
LanE	BAD714596<br>WP_008881432	GK0311<br>GK_RS02015
LanG	BAD74595<br>WP_004888811	GK0310<br>GK_RS02010

[1] The position of the gene annotated on the RefSeq genome differs from that predicted by antiSMASH 3.0. On the RefSeq genome, the position of the gene is (328676..329895), while the position predicted by antiSMASH 3.0 is (328679..329833).

Table S5. Annotation of the proteins coded by the lanthipeptide biosynthesis gene cluster of *G. thermodenitrificans* NG80-2. As described by NCBI, protein accession numbers with the WP_ prefix represent "non-redundant RefSeq protein records" that are "found in RefSeq genomes from multiple species". Locus-tags containing "RS" represent annotations of the RefSeq genome records, while the others were derived from the annotations of the original genome records.

**Protein coded by a member of the lanthipeptide gene cluster**	**Accession number**	**Locus-tag**
GeoAI	ABO65649<br>WP_008881441	GTNG_0265<br>GTNG_RS18005
GeoB	ABO65650<br>WP_011886696	GTNG_0266<br>GTNG_RS01590
GeoC	ABO65652<br>WP_011886697	GTNG_0268<br>GTNG_RS01600
GeoTI	ABO65651<br>WP_008881438	GTNG_0267<br>GTNG_RS01595
GeoK	ABO65654<br>WP_011886698	GTNG_0270<br>GTNG_RS01610
GeoR	ABO65653<br>WP_008881436	GTNG_0269<br>GTNG_RS01605
GeoI	ABO65655<br>WP_011886699	GTNG_0271<br>GTNG_RS01615
GeoF	ABO65658<br>WP_011886702	GTNG_0274<br>GTNG_RS01630
GeoE	ABO65657<br>WP_011886701	GTNG_0273<br>GTNG_RS01625
GeoG	ABO65656<br>WP_041264183	GTNG_0272<br>GTNG_RS01620 [1]

[1] The position of the gene annotated on the RefSeq genome differs from that annotated on the original genome and predicted by antiSMASH 3.0. On the RefSeq genome, the position of the gene is complement (309209..309997), while based on the original genome annotation, as well as the antiSMASH 3.0, the position of the gene is complement (309209..309823).

Table S6. The set of previously described lanthipeptides that have supported the predictions of antiSMASH.

Bacterial species, strain (lanthipeptide reference number)	Refseq genome accession number	Annotation of the predicted lanthipeptide		Matching lanthipeptide [1] (100% identical)	Supportive reference [2]
		Original genome record	RefSeq genome record		
Bacillus clausii KSM-K16	NC_006582	Unannotated	"Gallidermin/nisin family lantibiotic"	- [3]	NCBI
Bacillus megaterium QM B1551 (I-II)	NC_014023	"Antibiotic protein, putative"	"Gallidermin/nisin family lantibiotic"	- [3]	NCBI, and [31]
Bacillus subtilis BSn5	NC_014976	"Hypothetical"	"Hypothetical"	Subtilomycin	BAGEL BLAST, and [32,33]
Bacillus subtilis spizizenii TU-B-10	NC_016047	Unannotated	"Gallidermin/nisin family lantibiotic"	Entianin	NCBI, BAGEL BLAST, and [47]
Bacillus subtilis spizizenii W23	NC_014479	"Hypothetical"	"Gallidermin/nisin family lantibiotic"	Subtilin	NCBI, and BAGEL BLAST
Bacillus thuringiensis serovar IS5056 (I-V)	NC_020394	"Hypothetical"	"Hypothetical"	Lanthipeptides of thuricin 4A cluster	[31]
Bacillus thuringiensis YBT-1518 (I-II)	NC_022873	"Antibiotic protein, putative"	"Gallidermin/nisin family lantibiotic"	- [3]	NCBI
Geobacillus kaustophilus HTA426 (I)	NC_006510	"Lantibiotic precursor"	"Gallidermin/nisin family lantibiotic"	- [3]	NCBI, [15], and [34]
Geobacillus kaustophilus HTA426 (II)	NC_006510	"Lantibiotic precursor"	"Lantibiotic nisin-A"	- [3]	NCBI, and [15]
Geobacillus thermodenitrificans NG80_2	NC_009328	"Lantibiotic antimicrobial precursor peptide"	"Gallidermin/nisin family lantibiotic"	100% geobacillin I	NCBI, and [34]
Lactococcus lactis CV56	NC_017486	"Nisin precursor NisinA"	"Gallidermin/nisin family lantibiotic"	Nisin A	NCBI, BAGEL BLAST, [15], and [35]
Lactococcus lactis IO	NC_020450	"Lantibiotic antimicrobial precursor"	"Gallidermin/nisin family lantibiotic"	Nisin Z	NCBI, and BAGEL BLAST
Paenibacillus polymyxa E681	NC_014483	"Lantibiotic paenilan precursor peptide"	"Gallidermin family protein"	Paenilan	NCBI, and [36]
Staphylococcus aureus 11819-97 (I)	NC_017351	"Lantibiotic gallidermin"	"Gallidermin/nisin family lantibiotic"	BsaA2	NCBI, and BAGEL BLAST
Staphylococcus aureus Bmb9393 (I)	NC_021670	"Gallidermin/nisin family lantibiotic"	"Gallidermin/nisin family lantibiotic"	BsaA2	NCBI, and BAGEL BLAST
Staphylococcus aureus COL (I)	NC_002951	"Lantibiotic epidermin precursor EpiA"	"Gallidermin/nisin family lantibiotic"	BsaA2	NCBI, and BAGEL BLAST
Staphylococcus aureus ED133 (I)	NC_017337	"Hypothetical"	"Gallidermin/nisin family lantibiotic"	BacCH91	NCBI, BAGEL BLAST, and [81]
Staphylococcus aureus ED133 (II)	NC_017337	"Hypothetical"	"Gallidermin/nisin family lantibiotic"	- [3]	NCBI
Staphylococcus aureus LGA251 (I-II)	NC_017349	"Lantibiotic precursor"	"Gallidermin/nisin family lantibiotic"	- [3]	NCBI
Staphylococcus aureus M1 (I)	NC_021059	"Lanthionine precursor peptide LanA"	"Gallidermin/nisin family lantibiotic"	BsaA2	NCBI, and BAGEL BLAST
Staphylococcus aureus MSSA476 (I)	NC_002953	"Putative isochorismatase"	"Gallidermin/nisin family lantibiotic"	BsaA2	NCBI, and BAGEL BLAST
Staphylococcus aureus MW2 (I)	NC_003923	"Hypothetical protein, similar to gallidermin	"Gallidermin/nisin family lantibiotic"	BsaA2	NCBI, and BAGEL BLAST

		precursor”			
Staphylococcus aureus NCTC 8325 (I)	NC_007795	"Gallidermin superfamily epiA, putative"	"Gallidermin superfamily epiA, putative"	BsaA2	NCBI, and BAGEL BLAST
Staphylococcus aureus Newman (I)	NC_009641	"Lantibiotic precursor"	“Gallidermin/nisin family lantibiotic”	BsaA2	NCBI, and BAGEL BLAST
Staphylococcus aureus RF122 (I)	NC_007622	“Hypothetical”	“Gallidermin/nisin family lantibiotic”	BacCH91	NCBI, [15], and [81]
Staphylococcus aureus RF122 (II)	NC_007622	“Hypothetical”	“Gallidermin/nisin family lantibiotic”	- [3]	NCBI
Staphylococcus aureus T0131 (I)	NC_017347	"Lantibiotic"	“Gallidermin/nisin family lantibiotic”	BsaA2	NCBI, and BAGEL BLAST
Staphylococcus aureus TW20 (I)	NC_017331	“Lantibiotic precursor"	“Gallidermin/nisin family lantibiotic”	BsaA2	NCBI, and BAGEL BLAST
Staphylococcus aureus USA300 FPR3757 (I)	NC_007793	“Lantibiotic epidermin biosynthesis protein EpiA”	“Gallidermin/nisin family lantibiotic”	BsaA2	NCBI, and BAGEL BLAST
Staphylococcus aureus USA300_TCH1516 (I)	NC_010079	“Lantibiotic epidermin EpiA"	“Gallidermin/nisin family lantibiotic”	BsaA2	NCBI, and BAGEL BLAST
Staphylococcus aureus VC40 (I)	NC_016912	“Lantibiotic epidermin biosynthesis protein EpiA"	“Gallidermin/nisin family lantibiotic”	BsaA2	NCBI, and BAGEL BLAST
Staphylococcus aureus Z172 (I)	NC_022604	“Lanthionine precursor peptide LanA”	“Gallidermin/nisin family lantibiotic”	BsaA2	NCBI, and BAGEL BLAST
Streptococcus intermedius C270	NC_022237	“Hypothetical”	“Gallidermin/nisin family lantibiotic”	- [3]	NCBI
Streptococcus pasteurianus ATCC 43144	NC_015600	“Nisin U lantibiotic”	“Gallidermin/nisin family lantibiotic”	- [3]	NCBI
Streptococcus pyogenes MGAS6180	NC_007296	“Lantibiotic”	“Gallidermin/nisin family lantibiotic”	Streptin	NCBI, BAGEL BLAST, [15], and [84]
Streptococcus pyogenes MGAS9429	NC_008021	“Lantibiotic biosynthesis sensor protein”	“Gallidermin/nisin family lantibiotic”	Streptin	NCBI, BAGEL BLAST, and [84]
Streptococcus pyogenes MGAS10270	NC_008022	“Lantibiotic srtA precursor”	“Gallidermin/nisin family lantibiotic”	Streptin	NCBI, BAGEL BLAST, [15], and [84]
Streptococcus pyogenes MGAS10750	NC_008024	“Lantibiotic srtA precursor”	“Gallidermin/nisin family lantibiotic”	- [3]	NCBI, and [15]
Streptococcus suis JS14	NC_017618	Unannotated	“Gallidermin/nisin family lantibiotic”	Suicin 90-1330	NCBI, and [96]
Streptococcus suis SC070731	NC_020526	Unannotated	“Gallidermin/nisin family lantibiotic”	Suicin 90-1330	NCBI, and [96]

[1] Identity to experimentally verified lanthipeptide. [2] Supportive reference represents literature study(ies) where the respective lanthipeptide was identified, annotation(s) of the genome deposited in NCBI (RefSeq/GenBank), and/or the results of BAGEL BLAST. [3] (-) indicates that among experimentally verified lanthipeptides, there is not any that has 100% identity to the respective lanthipeptide.

S.2.3.1. Identification of* Bacillus-*associated lanthipeptide gene clusters*

S.2.3.1.1. *B. clausii* KSM-K16

The nucleotide (nt) sequence of the gene coding for the antiSMASH-predicted lanthipeptide (Table S1) is 100% identical to "ABC_RS22115" annotated on the RefSeq as coding for a class-I lanthipeptide (Table S2). Thus, the RefSeq annotation supports our analysis (Table S6). The core sequence of the predicted lanthipeptide was briefly mentioned by van Heel *et al*, 2016 [30]. The blastp analysis indicated that the predicted lanthipeptide is 56% identical to clausin (Table 2). We noticed that clausin has not been reported in BAGEL database; therefore, we recommend including it as a class-I lanthipeptide.

S.2.3.1.2. *B. megaterium* QM B1551

The aa sequences of the two antiSMASH-predicted lanthipeptides are identical, but the nt sequences of their two coding-genes are not (Table S1). The nt sequence of the gene coding for lanthipeptide (I) showed 100% identity to "BMQ_RS27575", and that coding for lanthipeptide (II) showed 100% identity to "BMQ_RS27580". These genes have been annotated in the RefSeq record as coding for class-I lanthipeptides (Table S2). Both lanthipeptides showed 56% identity to gallidermin as indicated by BAGEL BLAST (Table 2). It is noteworthy that using BAGEL3, Xin *et al*, 2015 identified one of the putative lanthipeptides [31] presented in the current study. Thus, the RefSeq annotation and results of Xin *et al*, 2015 support our analysis (Table S6).

S.2.3.1.3. *B. subtilis spizizenii* DSM 15029T (TU-B-10)

Our analysis has indicated points that are worth noting, as discussed below.

Using antiSMASH analysis, the gene coding for entianin was predicted (Table S1). In addition to the original and RefSeq genome sequence records of the strain, there is also a record on the entianin gene cluster that belongs to the same strain, separately deposited in NCBI under the accession number (HQ871873). We noticed that the entianin-coding gene has been annotated on the RefSeq as "GYO_RS39160" that codes for a class-I lanthipeptide. On the other hand, the coding gene in the entianin cluster record has been annotated and named as "*etnS*" coding for "EtnS" (AEK64494). The nt sequences of "GYO_RS39160" and "*etnS*" are identical and they both code for the same peptide. The two separate records related to the lanthipeptide resulted in having different tags/names for the same gene and its coded lanthipeptide. The entianin cluster (HQ871873) of the strain was sequenced by Fuchs *et al* and deposited in NCBI in 2011 [47] and the designation of the strain displayed for the record is DSM 15029^T. The antimicrobial activity of the entianin cluster has been experimentally proved [47]. On the other hand, the original genome (CP002905) was sequenced by Earl *et al* in 2012 [46], whereas the RefSeq genome was annotated in 2017, and the stated designation of the strain for both genome records is (TU-B-10). Accordingly, a re-evaluation of naming the gene and its lanthipeptide product in the RefSeq needs to be considered to match the description of the experimentally-verified entianin cluster (HQ871873). Moreover, since the RefSeq genome record shows only one of the designations of the strain; TU-B-10 while the record of the entianin cluster shows the other designation; DSM 15029^T, we recommend to present both designations in each record in order to avoid the confusion. Interestingly, the antibacterial activity of entianin has been experimentally confirmed; it is highly active against several Gram-positive bacteria [47].

S.2.3.1.4. *B. subtilis spizizenii* W23

The nt sequence of the gene coding for the antiSMASH-predicted lanthipeptide (Table S1) is 100% identical to "BSUW23_RS16845" annotated on the RefSeq as coding for a class-I

lanthipeptide (Table S2). The RefSeq annotation thus supports our analysis (Table S6). BAGEL BLAST indicated that the predicted lanthipeptide is 100% identical to subtilin (Table 2; Table S2) and 95% to entianin.

S.2.3.1.5. *B. thuringiensis* YBT-1518

The antiSMASH analysis indicated that the class-I lanthipeptide cluster coded on the chromosome of strain YBT-1518 has two putative genes coding for two different lanthipeptides. The nt sequence of the gene coding for the predicted lanthipeptide (I) (Table S1) is 100% identical to "YBT1518_RS19670", and that of the gene coding for lanthipeptide (II) is 100% identical to "YBT1518_RS19675". Both genes have been annotated on the RefSeq genome as coding for class-I lanthipeptides. The RefSeq annotation thus supports our analysis (Table S6). Based on the analysis using BAGEL BLAST, the predicted lanthipeptides I and II are 53% and 51% identical to gallidermin, respectively (Table 2).

S.2.3.2. Identification of Geobacillus*-associated lanthipeptide gene clusters*

S.2.3.2.1. *G. kaustophilus* HTA426

The nt sequence of the gene coding for the antiSMASH-predicted lanthipeptide (I) (Table S1) is 100% identical to "GK_RS18085" annotated on the RefSeq genome as coding for a class-I lanthipeptide. Therefore, the genome annotation supports our results (Table S6). BAGEL BLAST showed that lanthipeptide (I) is 91% identical to the experimentally characterized antimicrobially-active lanthipeptide; geobacillin I (ABO65649) produced by *G. thermodenitrificans* NG80-2 [34]. Garg *et al* 2012 reported the same lanthipeptide for strain HTA426 [34], and this in turn further supports our analysis (Table 2, Table S6).

S.2.3.3. Identification of Lactococcus*-associated lanthipeptide gene clusters*

S.2.3.3.1. *L. lactis* strains CV56 and IO-1

The nt sequence of the gene coding for the antiSMASH-predicted lanthipeptide of strain CV56 (Table S1) is 100% identical to "CVCAS_RS03115", and that coding for the predicted lanthipeptide of strain IO-1 (Table S1) is 100% identical to "LILO_RS03015". Both genes have been annotated in the RefSeq genome records as coding for class-I lanthipeptides. The RefSeq annotations thus support our analysis (Table S6). BAGEL BLAST showed that the predicted lanthipeptide of strain CV56 is 100% identical to nisin A (Table 2). Our results agree with those of Gao *et al*, 2011 [35] and Marsh *et al*, 2010 [15] (Table S6). On the other hand, BAGEL BLAST showed that the predicted lanthipeptide of strain IO-1 is 100% identical to nisin Z (Table 2).

S.2.3.4. Identification of Paenibacillus*-associated lanthipeptide gene clusters*

S.2.3.4.1. *P. polymyxa* E681

The nt sequence of the gene coding for the predicted lanthipeptide (Table S1) is 100% identical to "PPE_RS07020", which has been annotated on the RefSeq as coding for a class-I lanthipeptide (Table S2). The aa sequence of the characterized class-I lanthipeptide; named paenilan produced by strain E681 [36] is identical to that inferred in our study for the same strain (Table S2). This in turn supports our analysis (Table S6). The antibacterial activity of paenilan has been experimentally verified by Park *et al* [36].

S.2.3.5. Identification of Staphylococcus*-associated lanthipeptide gene clusters*

S.2.3.5.1. *S. aureus* 11819-97

The nt sequence of the gene coding for the antiSMASH-predicted lanthipeptide (I) (Table S1) is 100% identical to "MS7_RS09745" annotated on the RefSeq genome as coding for a class-I lanthipeptide. The annotation of the genome thus supports our analysis (Table S6). Moreover, BAGEL BLAST showed that lanthipeptide (I) is 100% identical to BsaA2 (Table 2). The antimicrobial activity of BsaA2 has been experimentally verified in other *S. aureus* strains by Daly *et al*, 2010 [8].

The nt sequence of the gene coding for the antiSMASH-predicted lanthipeptide (II) (Table S1) is 100% identical to "MS7_RS09750" annotated on the RefSeq genome as coding for a class-I lanthipeptide (Table S2). However, in RefSeq and consequently also in antiSMASH, the start codon has been translated into an incorrect aa in the presented aa sequence. The nt sequence presented in RefSeq and antiSMASH showed the first codon as "TTG", which codes for the aa leucine (L) and not the reported methionine (M). Therefore, we edited the start aa of the predicted lanthipeptide (II) into the correct one (L) (Table S1). BAGEL BLAST showed that lanthipeptide (II) is 83% identical to BsaA2 (Table 2).

S.2.3.5.2. *S. aureus* COL

The nt sequence of the gene coding for the antiSMASH-predicted lanthipeptide (I) (Table S1) is 100% identical to "SACOL_RS09635" annotated on the RefSeq genome as coding for a class-I lanthipeptide. The annotation of the genome thus supports our analysis (Table S6). Moreover, BAGEL BLAST showed that lanthipeptide (I) is 100% identical to BsaA2 (Table 2). Daly *et al* 2010 reported strain COL as a putative producer of BsaA2 [8].

The nt sequence of the gene coding for the antiSMASH-predicted lanthipeptide (II) (Table S1) is 100% identical to "SACOL_RS09645" annotated on the RefSeq genome as coding for a class-I lanthipeptide (Table S2). Again, we made the same correction of the aa as we did for lanthipeptide (II) of strain 11819-97 (Table S1). BAGEL BLAST showed that lanthipeptide (II) is 79% identical to BsaA2 (Table 2).

S.2.3.5.3. *S. aureus* ED133

The nt sequence of the gene coding for the antiSMASH-predicted lanthipeptide (I) (Table S1) is 100% identical to "SAOV_RS09475", and that of the other predicted gene coding for lanthipeptide (II) (Table S1) is 100% identical to "SAOV_RS09490". Both genes have been annotated on the RefSeq genome as coding for a class-I lanthipeptide (Table S2), which in turn, supports our analysis (Table S6). Moreover, BAGEL BLAST showed that lanthipeptide (I) is 100% identical to BacCH91 produced by *S. aureus* strain CH91 [81] (Table 2; Table S2). On the other hand, lanthipeptide (II) is 85% identical to BsaA2 (Table 2; Table S2). Only the core sequence of lanthipeptide (II) was very briefly mentioned by van Heel *et al*, 2016 and was inferred based on an *in silico* analysis [30].

S.2.3.5.4. *S. aureus* M1

The nt sequence of the gene coding for the antiSMASH-predicted lanthipeptide (I) (Table S1) is 100% identical to "BN843_RS09660" annotated on the RefSeq genome as coding for a class-I lanthipeptide. The annotation of the genome thus supports our analysis (Table S6). Moreover, BAGEL BLAST showed that lanthipeptide (I) is 100% identical to BsaA2 (Table 2).

The nt sequence of the gene coding for the antiSMASH-predicted lanthipeptide (II) (Table S1) is 100% identical to "BN843_RS09665" annotated on the RefSeq as coding for a class-I lanthipeptide (Table S2). Here as well, we edited the start aa of the predicted lanthipeptide (II) of

strain M1 into the correct one (L) instead of M (Table S1). BAGEL BLAST showed that lanthipeptide (II) is 83% identical to BsaA2 (Table 2).

S.2.3.5.5. *S. aureus* MSSA476

The nt sequence of the gene coding for the antiSMASH-predicted lanthipeptide (I) (Table S1) is 100% identical to "SAS1746" which has been annotated on the RefSeq genome as coding for a class-I lanthipeptide (Table S2). The RefSeq genome annotation thus supports our analysis (Table S6). BAGEL BLAST showed that lanthipeptide (I) is 100% identical to BsaA2 (Table 2). Daly *et al*, 2010 reported strain MSSA476 as a putative producer of BsaA2 [8].

The nt sequence of the gene coding for the antiSMASH–predicted lanthipeptide (II) (Table S1) is 100% identical to "SAS_RS09300" annotated on the RefSeq genome as coding for a class-I lanthipeptide. Here as well, we edited the start aa of the predicted lanthipeptide (II) of strain MSSA476 into the correct one (L) instead of M (Table S1). BAGEL BLAST showed that lanthipeptide (II) is 83% identical to BsaA2 (Table 2).

S.2.3.5.6. *S. aureus* MW2

The nt sequence of the gene coding for the antiSMASH-predicted lanthipeptide (I) (Table S1) is 100% identical to "MW_RS09420" annotated on the RefSeq as coding for a class-I lanthipeptide. Therefore, the RefSeq annotation supports our analysis (Table S6). BAGEL BLAST showed that lanthipeptide (I) is 100% identical to BsaA2 (Table 2). Daly *et al*, 2010 reported strain MW2 as a putative producer of BsaA2 [8].

The nt sequence of the gene coding for the antiSMASH-predicted lanthipeptide (II) (Table S1) is 100% identical to "MW_RS09425" annotated on the RefSeq as coding for a class-I lanthipeptide. Here as well, we edited the start aa of the predicted lanthipeptide (II) of strain MW2 into the correct one (L) instead of M (Table S1). BAGEL BLAST showed that lanthipeptide (II) is 83% identical to BsaA2 (Table 2).

S.2.3.5.7. *S. aureus* NCTC 8325

The nt sequence of the gene coding for the antiSMASH-predicted lanthipeptide (I) (Table S1) is 100% identical to "SAOUHSC_01953" annotated on the RefSeq genome as coding for a class-I lanthipeptide. The annotation of the genome thus supports our analysis (Table S6). BAGEL BLAST showed that lanthipeptide (I) is 100% identical to BsaA2 (Table 2). Daly *et al*, 2010 reported strain NCTC 8325 as a producer of BsaA2 [8].

S.2.3.5.8. *S. aureus* RF122

The nt sequence of the gene coding for the antiSMASH–predicted lanthipeptide (I) (Table S1) is 100% identical to "SAB_RS08990" annotated on the RefSeq genome as coding for a class-I lanthipeptide (Table S2), which thus supports our analysis (Table S6). Based on the analysis using BAGEL BLAST, lanthipeptide (I) is 100% identical to BacCH91, and 81% identical to BsaA2. Moreover, our results agree with the findings of Marsh *et al*, 2010 [15].

The nt sequence of the gene coding for the antiSMASH-predicted lanthipeptide (II) (Table S1) is 100% identical to "SAB_RS08995" annotated on the RefSeq as coding for a class-I lanthipeptide (Table S2). Thus, the RefSeq annotation supports our analysis (Table S6). Based on the analysis using BAGEL BLAST, lanthipeptide (II) is 87% identical to BsaA2. Daly *et al*, 2010 also reported strain RF122 as a producer of variants of Bsa [8].

S.2.3.5.9. *S. aureus* T0131

The nt sequence of the gene coding for the antiSMASH-predicted lanthipeptide (I) (Table S1) is 100% identical to "SAT0131_RS09535" annotated on the RefSeq genome as coding for a class-I lanthipeptide. The annotation of the genome thus supports our analysis (Table S6). BAGEL BLAST showed that lanthipeptide (I) is 100% identical to BsaA2 (Table 2).

The nt sequence of the gene coding for the antiSMASH-predicted lanthipeptide (II) (Table S1) is 100% identical to "SAT0131_RS09540" annotated on the RefSeq genome as coding for a class-I lanthipeptide. Here as well, we edited the start aa of the predicted lanthipeptide (II) of strain T0131 into the correct one (L) instead of M (Table S1). BAGEL BLAST showed that lanthipeptide (II) is 83% identical to BsaA2 (Table 2).

S.2.3.5.8.10. *S. aureus* strains USA300 FPR3757 and USA300_TCH1516

The nt sequence of the gene coding for the antiSMASH-predicted lanthipeptide (I) of strain USA300_FPR3757 (Table S1) is 100% identical to "SAUSA300_RS09670". For strain USA300_TCH1516, the nt sequence of the gene coding for the predicted lanthipeptide (I) (Table S1) is 100% identical to "USA300HOU_RS09705". Each of "SAUSA300_RS09670" and "USA300HOU_RS09705" has been annotated on the RefSeq genome of the respective strain as coding for a class-I lanthipeptide. The annotation of the genome thus supports our analysis (Table S6). BAGEL BLAST showed that each of the predicated lanthipeptides is 100% identical to BsaA2 (Table 2). Daly *et al* (2010) reported that the two strains are putative producers of BsaA2 [8].

The nt sequence of the gene coding for the antiSMASH-predicted lanthipeptide (II) of strain USA300 FPR3757 (Table S2) is 100% identical to "SAUSA300_RS09675", and that of the gene coding for the predicted lanthipeptide (II) of strain "USA300_TCH1516" (Table S2) is identical to "USA300HOU_RS09710". Here as well, we edited the start aa of the predicted lanthipeptide (II) of both strains into the correct one (L) instead of M (Table S1). BAGEL BLAST indicated that lanthipeptide (II) of each of the strains is 83% identical to BsaA2 (Table 2).

S.2.3.5.8.11. *S. aureus* Z172

The nt sequence of the gene coding for lanthipeptide (I) (Table S1) is 100% identical to "SAZ172_RS09800" annotated on the RefSeq genome as coding for a class-I lanthipeptide. The annotation of the genome thus supports our analysis (Table S6). BAGEL BLAST showed that lanthipeptide (I) is 100% identical to BsaA2 (Table 2).

The nt sequence of the antiSMASH-predicted lanthipeptide (II) (Table S1) is 100% identical to "SAZ172_RS09810" annotated on the RefSeq genome as coding for a class-I lanthipeptide (Table S2). Here as well, we edited the start aa of the predicted lanthipeptide (II) of strain Z172 into the correct one (L) instead of M (Table S1). BAGEL BLAST showed that lanthipeptide (II) is 83% identical to BsaA2 (Table 2).

S.2.3.5.8.12. other strains

The aa sequence of lanthipeptide (I) is identical in strains Bmb 9393, Newman, TW20, and VC40 (Table S1). The coding genes have been annotated in the RefSeq genome record of strain Bmb 9393, as "SABB_RS10200"; strain Newman as "NWMN_RS09635"; strain TW20, as "SATW20_RS09830"; and strain VC40, as "SAVC_RS08915". Each of the genes has been annotated as coding for a class-I lanthipeptide. Therefore, the genome annotations support our results (Table S6). Moreover, the predicted lanthipeptides showed 100% identity to the BsaA2, as indicated by BAGEL BLAST (Table 2).

The aa sequence of lanthipeptide (II) in all these strains is identical (Table S1). The coding genes have been annotated in the RefSeq genome records as coding for class-I lanthipeptides. The

nt sequence of the predicted gene coding for lanthipeptide (II) of strain Bmb 9393 is 100% identical to "SABB_RS10205", that of strain Newman is 100% identical to "NWMN_RS09640", that of strain TW20 is 100% identical to "SATW20_RS09835", and that of strain VC40 is 100% identical to "SAVC_RS08920". Here as well, we edited the start aa of the predicted lanthipeptide (II) of each of these strains into the correct one (L) instead of M (Table S1). The predicted lanthipeptides showed 83% identity to the BsaA2, as indicated by BAGEL BLAST (Table 2).

Furthermore, our analysis indicated that *S. aureus* strain LGA251 harbours a class-I lanthipeptide cluster coding for two different lanthipeptides. The putative coding genes of lanthipeptides (I) and (II) (Table S1) have been annotated on the RefSeq genome as "SARLGA251_RS09195" and "SARLGA251_RS09200", each of which has been annotated as coding for a class-I lanthipeptide. Therefore, RefSeq annotations support our analysis (Table S6). BAGEL BLAST indicated that lanthipeptides (I) and (II) showed 81% and 85% identity, respectively, to the BsaA2 of *S. aureus* (Table 2).

S.2.3.6. Identification of Streptococcus-associated lanthipeptide gene clusters

S.2.3.6.1. *S. intermedius* C270

The nt sequence of the gene coding for the antiSMASH-predicted lanthipeptide (Table S1) is 100% identical to "SII_RS05025" annotated on the RefSeq as coding for a class-I lanthipeptide (Table S2). The RefSeq annotation, in turn, supports our analysis (Table S6). Based on the analysis using BAGEL BLAST, the predicted lanthipeptide is 81% identical to Nisin F which is produced by *L. lactis* (Table 2; Table S2).

S.2.3.6.2. *S. pasteurianus* ATCC 43144

The nt sequence of the gene coding for the lanthipeptide (Table S1) is 100% identical to "SGPB_RS10680" annotated on the RefSeq as coding for a class-I lanthipeptide. The genome annotation, thus, supports our analysis (Table S6) and also confirms the results of Lin *et al*, 2011. Based on the analysis using BAGEL BLAST, the predicted lanthipeptide is 91% similar to nisin U (Table 2). These results indicate that the antimicrobial potential of the lanthipeptide in strain ATCC 43144 should be of interest to investigate.

S.2.3.6.3. *S. pyogenes* MGAS9429

The nt sequence coding for the antiSMASH-predicted lanthipeptide (Table S1) is 100% identical to "MGAS9429_RS04535" annotated on the RefSeq genome as coding for a class-I lanthipeptide (Table S2). This in turn supports our analysis (Table S6). Moreover, BAGEL BLAST indicated that the predicted lanthipeptide is 100% identical to streptin (Table 2). Therefore, we suggest considering *S. pyogenes* strain MGAS2096 as another potential streptin-producer.

S.2.3.6.4. Other strains of *S. pyogenes*

The aa sequences of the lanthipeptides of strains MGAS6180, MGAS10270 and MGAS10750 are identical (Table S1). The nt sequence of the gene coding for the lanthipeptide of strain MGAS6180 (Table S1) is 100% identical to "M28_RS03970". That of the gene coding for the lanthipeptide of strain MGAS10270 (Table S1) is 100% identical to "MGAS10270_RS04550". Finally, the nt sequence of the gene coding for the lanthipeptide of strain MGAS10750 (Table S1) is identical to "MGAS10750_RS04710". These 3 genes have been annotated in the RefSeq genome records as coding for class-I lanthipeptides. Therefore, our results are supported by the annotation of the genome records of the three strains (Table S6). BAGEL BLAST indicated that the the lanthipeptides of strains MGAS6180 and MGAS10270 are 100% identical to streptin, while

that of strain MGAS10750 is 98% identical to streptin (Table2). The results of antiSMASH analysis agree with the findings of Marsh *et al*, 2010 [15].

S.2.3.6.5. *S. suis* strains JS14 and SC070731

The nt sequences of the gene coding for the antiSMASH-predicted lanthipeptide of strain JS14, and that coding for the lanthipeptide of strain SC070731 are identical (Table S1). The coding genes have been annotated as "SSUJS14_RS10730" on the RefSeq genome of strain JS14, and as "NJAUSS_RS10650" on that of strain SC070731. Each of these genes codes for a class-I lanthipeptide. Therefore, the annotation of the RefSeq genome supports our analysis (Table S6). Each of the predicted lanthipeptides is 100% identical to suicin 90-1330 produced by *S. suis* strain 90-1330 [96] (Table 2). We noticed that suicin 90-1330 has not been reported in BAGEL databases. Since the lanthipeptide production potential of strains JS14 and SC07073 has not been investigated, we suggest the two strains as potential lanthipeptide producers.

* The section number corresponds to that in the main text but preceded with an "S." for "supplementary".

Additional files

- Software source code is available at http://130.235.46.10/Lanthipeptides/Software/
- Graphical illustrations of the antiSMASH output are available at http://130.235.46.10/Lanthipeptides/
